# Dual-Lifetime Referencing (*t*-DLR) Optical Fiber Fluorescent pH Sensor for Microenvironments

**DOI:** 10.3390/s23218865

**Published:** 2023-10-31

**Authors:** Wan-Har Chen, Evelyn Armstrong, Peter W. Dillingham, Stephen C. Moratti, Courtney Ennis, Christina M. McGraw

**Affiliations:** 1 Department of Chemistry, University of Otago, Dunedin 9054, New Zealand; whchen88@msn.com (W.-H.C.); sc.moratti@googlemail.com (S.C.M.); courtney.ennis@otago.ac.nz (C.E.); 2 NIWA/University of Otago Centre for Oceanography, Department of Marine Science, University of Otago, Dunedin 9054, New Zealand; evelyn.armstrong@otago.ac.nz; 3 Department of Mathematics and Statistics, University of Otago, Dunedin 9054, New Zealand; peter.dillingham@otago.ac.nz; 4 Coastal People Southern Skies Centre of Research Excellence, University of Otago, Dunedin 9054, New Zealand

**Keywords:** optical fiber fluorescent pH sensor, dual-layer sensing film, time-domain dual-lifetime referencing, ocean acidification, marine microenvironments, inverse calibration

## Abstract

The pH behavior in the μm to cm thick diffusion boundary layer (DBL) surrounding many aquatic species is dependent on light-controlled metabolic activities. This DBL microenvironment exhibits different pH behavior to bulk seawater, which can reduce the exposure of calcifying species to ocean acidification conditions. A low-cost time-domain dual-lifetime referencing (*t*-DLR) interrogation system and an optical fiber fluorescent pH sensor were developed for pH measurements in the DBL interface. The pH sensor utilized dual-layer sol-gel coatings of pH-sensitive iminocoumarin and pH-insensitive Ru(dpp)_3_-PAN. The sensor has a dynamic range of 7.41 (±0.20) to 9.42 ± 0.23 pH units (95% CI, T = 20 °C, *S* = 35), a response time (*t*_90_) of 29 to 100 s, and minimal salinity dependency. The pH sensor has a precision of approximately 0.02 pH_T_ units, which meets the Global Ocean Acidification Observing Network (GOA-ON) “*weather*” measurement quality guideline. The suitability of the *t*-DLR optical fiber pH sensor was demonstrated through real-time measurements in the DBL of green seaweed *Ulva* sp. This research highlights the practicability of optical fiber pH sensors by demonstrating real-time pH measurements of metabolic-induced pH changes.

## 1. Introduction

The oceans play a significant role in climate mitigation by absorbing approximately 30% of anthropogenic CO_2_ emissions [1], causing the pH of the ocean’s surface to decline by ca. 0.1 pH units [2,3,4,5]. Ocean acidification (OA) results in a shift of the carbonate equilibria, and the reduction in ${[\mathrm{CO}}_{3}^{2-}]$ has made it challenging for marine calcifying species to maintain their shells and skeletons [6,7,8,9]. Numerous laboratory experiments [10,11,12,13,14], modeling studies [15,16,17], and field observations [18,19] strongly suggest that OA will impact marine biodiversity and alter ecosystem processes [4,5,6,9,20].

OA laboratory studies have shown that photosynthetic marine species, such as macroalgae [13,14,21,22], can biologically modify the local chemical microenvironment within their discrete μm to cm thick diffusion boundary layer (DBL) [10,23,24]. Nutrient exchange between the organism and the bulk environment is via a diffusion process [10]. Metabolic gases and ions within the DBL result in a concentration gradient that forms a distinct microenvironment and shows a different pH behavior compared to bulk seawater. The pH of the seawater in this region increases under light irradiation due to photosynthesis and reduces in the dark due to respiration [13,14]. A high thickness and concentration gradient of DBL has been shown to reduce the OA effect on vulnerable calcifying species [10,11,12]. The thickness and chemical composition of the DBL are governed by external environmental factors such as flow rate and light conditions [10,21,25].

To understand the impact of OA on marine species, pH sensors capable of making measurements in microenvironments are required. The studies of OA’s impact on marine species [26,27] typically utilize miniaturized electrochemical sensors [23,28] to measure the changes in pH, oxygen concentration, and thickness gradient of the DBL [23,24,29,30,31]. These sensors are fragile and prone to electromagnetic field interference. The commercially available microsensors are limited to a few specialized suppliers [32,33], and have a high replacement cost (hundreds of USD). There is a demand for small-sized sensors that are cost-effective and possess remote and real-time monitoring properties [34,35,36].

Ongoing research efforts are dedicated to exploring innovative approaches to create miniaturized, more durable, and cost-effective electrochemical pH sensors. The comprehensive review of the newly emerging pH sensor materials by Avolio et al. [37] provides an up-to-date overview of various pH measurement techniques. The review covers established electrochemical pH-sensing methods as well as recent advancements in a broad spectrum of sensor materials, such as inorganic, organic, and nano-engineered devices. These studies hold promise for various environmental monitoring applications and are instrumental in informing our approach to optical fiber pH sensor development, particularly in addressing the unique challenges posed by marine microenvironments.

In recent years, significant research has been dedicated to the development of optical pH sensing [38]. There are a great number of optical sensing approaches with numerous specific features, of which planar sensors [39,40] and optical fiber sensors [34,36,41] are well-established. Many state-of-the-art optical sensors are based on nanoparticles with pH-sensing properties [42,43]. The small dimensions of nanoparticles significantly minimize the diffusion passage of analytes, thus having a fast pH response. At present, these nanoparticles are mainly used for in vivo intracellular pH-sensing applications [44,45], although their practicality can be limited by cytotoxicity and the complexity of biological delivery systems.

Optical fiber sensors are insensitive to electromagnetic field interference. They have the capability of remote and continuous sensing, and their microstructures offer minimal invasiveness [46,47]. In addition, optical fiber pH sensors have low sensor fabrication costs, offering an alternative solution to pH measurements in marine microenvironments [48]. Typically, optical fiber pH sensors are based on fibers coated with a pH-sensitive indicator immobilized in a polymeric or sol-gel-derived matrix. The interaction of evanescent waves (EW) with the pH-sensitive layer enables the determination of pH in the surrounding medium [49,50].

Optical fiber fluorescent pH sensors are well-suited for pH measurements in challenging locations such as marine environments [48]. The dual-lifetime referencing (DLR) approach uses the ratio of two luminophores with distinct differences in their decay time: a pH-sensitive fluorescent indicator and a pH-insensitive phosphorescent dye reference. pH is derived from the ratio of the fluorescent intensity determined either in the time (*t*-DLR) or frequency domain (*f*-DLR) [51]. This approach allows the signal to be referenced internally, independent of fluorophore concentration and variations in excitation intensity [39,52,53,54,55]. Nonetheless, current DLR measurements require sophisticated and costly devices. The objective of this study was to develop a *t*-DLR interrogation system using fluorescent optical fiber pH sensors for marine environments. The *t*-DLR instrumentation utilized low-cost electronic components and commercially available optical elements, reducing the cost of the system from ca. USD 12,000–16,000 USD to USD 3400 USD (Table S1).

To facilitate the use of DLR in marine environments, suitable pH-sensitive fluorophores (pH 7.5 to 9.0) are required. Most fluorophores that meet this requirement have a hydrophilic nature, are sensitive to ionic strength (IS), and have a high material cost (Table S2). In contrast, iminocoumarin overcomes most of these disadvantages [56,57,58,59] and it was synthesized and used as the pH-sensitive indicator in this study. The *t*-DLR application for pH measurement also requires a pH-insensitive reference with long fluorescence lifetime. Tris(4,7-diphenyl-1,10-phenanthroline) ruthenium(II) dichloride complex (Ru(dpp)_3_) was chosen due to its good photostability and long lifetime. Encapsulation of Ru(dpp)_3_ in solid matrices and as nanospheres [60,61,62] has been widely used in the development of oxygen sensors [63,64], temperature sensors [65], and applied as the pH-insensitive reference in DLR pH sensors [55,66,67,68]. Here, Ru(dpp)_3_ was encapsulated into a low-gas-permeable polymer, polyacrylonitrile (PAN) [60,61,62,69], to prevent oxygen quenching.

The effect of photobleaching is a factor that cannot be offset using DLR [38]. Studies have shown that immobilization of organic dyes in a sol-gel matrix greatly improves the photostability of the dye molecules [70,71,72]. The sol-gel silica network imposed a caging effect on the dye molecule, hindering the intermolecular motions and reducing diffusion of triplet quenchers, thereby minimizing the reactive singlet oxygen species. Both factors can greatly reduce the photodegradation of the dye molecule.

The pH sensor in this work was based on EW sensing. In this application, iminocoumarin and Ru(dpp)_3_-PAN particles were entrapped in an optimized sol-gel matrix of tetraorthosilicate (TEOS) and dimethyldiethoxy silane (DDS) [48] and then directly coated onto the optical fiber core as a dual-layer pH-sensing film. This dual-layer pH sensor had a p*K*_a_’ of 8.66 (T = 20 °C, *S* = 35), was insensitive to salinity, and had negligible dye leaching. The pH sensor had an accuracy of 0.023 pH units and a precision of 0.021 pH units, which conforms to the GOA-ON “*weather*” measurement quality guideline [73] for the identification of relative spatial patterns, and short-term variation in biological and environmental studies. The suitability of the low-cost *t*-DLR optical fiber pH sensor was demonstrated through the detection of metabolic-induced pH changes in the DBL of the green seaweed *Ulva* sp. [74].

## 2. Materials and Methods

### 2.1. Chemicals and Reagents

All chemicals were reagent grade and used without further purification. Solutions were prepared using Milli-Q water (Millipore S.A., Molsheim, France, 18.2 Ω), and all chemicals were purchased from Sigma-Aldrich (St. Louis, MO, USA) unless otherwise stated. 4-(diethylamino)salicylaldehyde (98%), (2-benzimidazolyl)acetonitrile (97%), piperidine, and dry methanol (MOLECULAR SIEVES, 3Å, CHEM store, Dunedin, New Zealand) were used to synthesize iminocoumarin. Ru(dpp)_3_ (Santa Cruz Biotechnology, Dallas, TX, USA), PAN (MW 150,000), and N,N’-dimethylformamide (DMF) (Ajax Finechem, Seven Hills, NSW, Australia) were used to prepare Ru(dpp)_3_-PAN particles. Trifluoroacetic acid-d (TFA-d) and dimethyl sulfoxide-d_6_ (DMSO-d_6_) were used in ^1^H and ^13^C NMR spectroscopy.

Sol-gel matrices were prepared using TEOS and DDS (97%), Triton™ X-100 (Romil, Cambridge, UK), ethanol, and 0.1 M hydrochloric acid (HCl, Milli-Q). Artificial seawater (ASW) was prepared based on Roy et al. [75], and Tris buffers in ASW (pH_T_ range 8.16 to 9.45) using pH total scale (pH_T_) [76] were prepared according to Pratt [77] with Tris(hydroxymethyl)aminomethane (99.8%, BDH). The pH reference was a pH electrode calibrated using Tris buffer (pH_T_ = 8.092 at 25 °C), prepared by the NIWA/University of Otago Research Centre for Oceanography [78]. Aqueous solutions of 0.2 M HCl and 0.2 M NaOH with IS adjusted to 0.7 M with NaCl were used in the pH titration.

### 2.2. Iminocoumarin Synthesis and Encapsulation of Ru(dpp)_3_ in PAN

Iminocoumarin was synthesized according to a previously described method [79,80]. The structure and purity of the synthesized indicator were characterized through HR-MS (Shimadzu LCMS-9030) (Figure S1), ^1^H (Figure S2), and ^13^C NMR spectroscopy (Figure S3). The preparation of Ru(dpp)_3_-PAN was a modification of the procedure of Borisov et al. [61]. The additional steps included centrifugation of the suspension, followed by dialysis of the slurry in Milli-Q for 3 days, and then particles of Ru(dpp)_3_-PAN were obtained by freeze-drying for 48 h. The ^1^H NMR of Ru(dpp)_3_-PAN showed no DMF peaks [81] at 7.95, 2.89, and 2.75 ppm (Figure S4). The particle size of Ru(dpp)_3_-PAN was analyzed using dynamic light scattering (Figure S5), indicating that highly agglomerated particles (zeta potential −5.52 ± 0.1 mV) were obtained via a freeze-drying process.

The properties of the fluorescence spectra of iminocoumarin, Ru(dpp)_3_, and Ru(dpp)_3_-PAN immobilized in sol-gel (Ru(dpp)_3_-PAN-SG)—absorption (ex), emission (em), extinction coefficient (ε), absolute fluorescence quantum yield (Φ), and fluorescence lifetime (τ)—were evaluated (FS5 and FLUORACLE software version 1.9.4, Edinburgh Instruments, Livingston, UK). Fluorescence spectra of iminocoumarin showed the indicator was pH-dependent (Figure S6). The brightness of iminocoumarin satisfied the requirement of fluorescent sensing using thin film (Φ × ε >20,000) [38]. The PAN and sol-gel shielding layers prevented Ru(dpp)_3_ oxygen quenching (Φ = 40.3%), compared to the un-shielded Ru(dpp)_3_ (Φ = 6.17%). Immobilization of both iminocoumarin and Ru(dpp)_3_-PAN showed an absorbance band (λ = 465 ± 15 nm), and two emission peaks (λ = 530 and 610 nm) (Figure S7). The excitation overlapping region allowed a single light source to excite both luminophores simultaneously. The fluorescence properties are summarized in Table 1.

### 2.3. Dual-Layer Sol-Gel pH-Sensing Coating

The optical fiber pH sensor was fabricated from a 12 cm long fiber (FT400UMT, Thorlabs). The sol-gel matrix and the 2 cm pH-sensing region were prepared according to previous methods [31]. The pH sensor was configured as two layers of sol-gel coating, the first (inner) layer containing Ru(dpp)_3_-PAN and the second (outer) layer containing iminocoumarin (Table S3).

To minimize agglomeration, Ru(dpp)_3_-PAN sol was sonicated before dip-coating and agitated (2200 rpm) during the dip-coating process. The Ru(dpp)_3_-PAN sol was applied as the first coat onto the fiber core. After 3 days, the iminocoumarin sol was dip-coated over the Ru(dpp)_3_-PAN sol-gel coating to avoid redissolution of the Ru(dpp)_3_-PAN layer. The sensing film thickness (6.6 μm) was determined microscopically (Leica DFC295) (Figure S8).

### 2.4. The t-DLR Interrogation System

#### 2.4.1. pH Measurement Based on the *t*-DLR Principle

**Figure 1:**
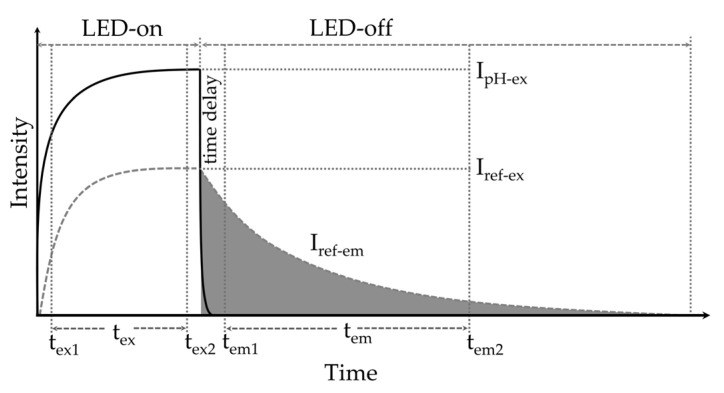
*t*-DLR schematic. The excitation intensity (I_ex_) during the LED-on (t_ex_) consists of both pH-sensitive (**▬,** I_pH-ex_) and pH-insensitive dye (⁃⁃⁃, I_ref-ex_) intensities. The shaded emission intensity during the LED-off (t_em_) is assumed to be solely the pH-insensitive dye (I_ref-em_).

The acquisition of fluorescence excitation and emission intensity was carried out during the excitation period (t_ex_) when the light was switched on (LED-on), and the fluorescence decay period (t_em_) when the light was switched off (LED-off). The ratio of the excitation and emission-integrated areas was used to determine the pH of the analyte solution (Figure 1).

During the LED-on pulse time (t_ex_), the excitation intensity (I_ex_) (Equation (1)) consisted of the sum of intensities from both the short-lived pH-sensitive iminocoumarin (I_pH-ex_) and the long-lived pH-insensitive Ru(dpp)_3_-PAN (I_ref-ex_).

I_ex_ = I_pH-ex_ + I_ref-ex_ (1)

Due to the rapid decay of the pH-sensitive dye, the emission intensity (I_em_) during the LED-off (t_em_) period is assumed to consist exclusively of the long-lived pH-insensitive Ru(dpp)_3_-PAN. To ensure the complete decay of the iminocoumarin, and to exclude the LED “settling time” which occurs as the current builds up and discharges during the switch-on and switch-off periods, a time delay (1.1 μs) between the end of t_ex_ and the start of t_em_ was applied. The intensities I_ex_ and I_ref-em_ over the selected LED-on (t_ex_) and LED-off (t_em_) duration, respectively, were integrated using a MATLAB trapezoidal algorithm. The integrated intensities over the excitation and emission periods, D_ex_ and D_em_, were then used to calculate the ratio, *R*, of the two periods:

(2) $$R=\frac{\int_{t_{\mathrm{ex}1}}^{t_{\mathrm{ex}2}}(I_{\mathrm{pH}-\mathrm{ex}+I_{\mathrm{ref}-\mathrm{ex}}})\;d_{t}}{\int_{t_{\mathrm{em}1}}^{t_{\mathrm{em}2}}I_{\mathrm{ref}-\mathrm{em}}d_{t}}=\frac{D_{\mathrm{ex}}}{D_{\mathrm{em}}}$$

#### 2.4.2. *t*-DLR instrumentation and Dual-Layer pH Sensor Response to pH Variation

The *t*-DLR signal interrogation system (Figure S9) used custom-made electronic elements assembled with commercial optical and electronic components. A lens tube spacer (04ETS1-S1-1L, Unice, Taoyuan City, Taiwan) joined the photomultiplier tube (PMT 9220, Hamamatsu, Hamamatsu City, Japan) through the light-tight PMT housing (PXT1/M, Thorlabs, Saint-Laurent, QC, Canada) to a fluorescence filter cube (DFM1/M, Thorlabs, Newton, NJ, USA), which was used to mount fluorescent filters: an excitation filter (ET470/40x GFP, Chroma, Bellows Falls, VT, USA), a dichroic mirror (DMLP490R, Thorlabs, USA), and an emission filter (ET510LP, Chroma). The optical pathway was formed using fiber optic patch cables (FT400UMT, Thorlabs, USA), linking the fiber-coupled light-emitting diode (LED, λ_470nm_) (M470F3, Thorlabs, Dortmund, Germany), filter cube, and pH sensor.

An arbitrary waveform generator (AWG) (FY8300S, FeelElec Technology, Zhengzhou, China) in conjunction with a custom-built LED driver (Figure S10) pulsed the LED at 20 kHz (10 μs on, 40 μs off) to generate the excitation and emission periods. The detected fluorescence signals were amplified via a custom-built 2-stage PMT amplifier (Figure S11). A digital oscilloscope (PicoScope) (2406B, Pico Technologies, St Neots, UK) set the trigger conditions, enabling the repetitive signals to be stabilized and captured as waveforms using PicoScope^®^ 6—PC Oscilloscope software (version: 6.14.61.6219, Pico Technology Ltd., St Neots, UK).

**Figure 2:**
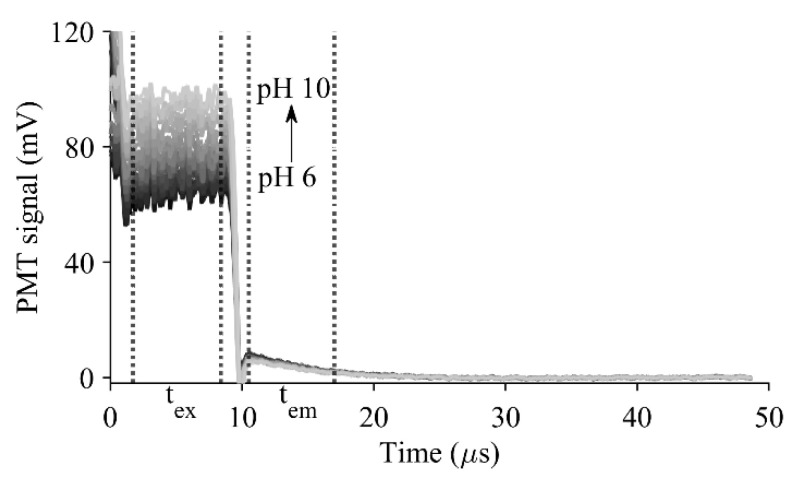
The averaged pulsed signals of the dual-layer sensor using the *t*-DLR system. LED-on (t_ex_) and LED-off (t_em_) regions are denoted with (**…**).

The acquisition time was 1.5 s per pH reading, which comprised 6250 data points (8 ns sample interval), 30 PicoScope data files, and 1000 × 30 cycles (Figure S12a). To improve the signal-to-noise ratio (SNR), the on–off cycles were averaged as a pulse wave (1 × 30,000 cycles) (Figure S12b). The background signal obtained from a sol-gel-coated optical fiber without luminophores was subtracted from the measured signals (Figure S12c), enabling the *R* ratio calculation. Following averaging and baseline adjustment, the excitation and emission profile of the luminophores were revealed as a pulsed signal. The intensity of the excitation period (t_ex_) increased with the increase in pH, while the intensity of the decay curve during the emission period (t_em_) remained unchanged with pH variations (Figure 2).

### 2.5. Characterization of the Optical Fiber Fluorescent pH Sensor

Photostability, indicator leaching, pH sensor usable lifetime, and response time of the pH sensor were investigated using a reflection probe (RP29, Thorlabs), a UV-Vis spectrometer (STS-VIS, Ocean Optics, Orlando, FL, USA), and the fiber-coupled LED. A LabVIEW interface (National Instruments) was used to record the UV-Vis intensity spectra (acquired every 0.22 min), pH electrode readings, and temperature. All other experiments were conducted at 20 ± 0.1 °C in ASW (*S* = 35), using the *t*-DLR interrogation system.

#### 2.5.1. Photostability and Indicator Leaching of the pH Sensor

To investigate the pH sensor signal drift due to photobleaching, three optical fiber sensors were fabricated: iminocoumarin, Ru(dpp)_3_-PAN, and the dual-layer sol-gel coating. The sensors were held in a dark and dry chamber and separately exposed to the LED for four hours. The optical intensity difference over the experimental period was used to derive the sensor’s intensity drift over time.

To investigate the dual-layer pH sensor signal drift due to indicator leaching, the pH sensor was held in ASW (approximately pH_T_ = 8) in a dark chamber. The intensity at time 0, 5.5, and 22 h was used to determine the sol-gel-immobilized indicators’ resistance to leaching in a seawater environment.

#### 2.5.2. The Usable Lifetime of the pH Sensor

To investigate the applicable lifetime of the dual-layer pH sensor, an experimental period of approximately 150 min was used. This chosen time period would represent an estimated 12 h continuous operation of the pH sensor, using *t*-DLR interrogation at a frequency of 20 kHz (20% duty cycle). This experiment was carried out in a dark environment, and the sensor was held in ASW (*S* = 35, T = 20 °C) and continuously exposed to the LED light (λ = 470 nm). The pH_T_ of the ASW was repeatedly increased and decreased between pH_T_ 6.6 and 9.8 using 0.2 M HCl and 0.2 M NaOH solutions (IS = 0.7 M). The pH_T_ readings of an ASW Tris buffer-calibrated pH electrode were used as the reference. The optical signal of the pH sensor in ASW was acquired every 0.22 min (integration time = 250 ms, average = 50). The optical spectra of the pH sensor were normalized against the average intensity between wavelengths 338 and 420 nm, where the pH sensor has no response to pH changes. Subsequently, the normalized intensity of the pH sensor at wavelengths of iminocoumarin was plotted against time.

#### 2.5.3. pH Sensor Response Time

The sensor response time is an important characteristic of optical sensors since it is influenced by the properties of the thin film, the entrapped indicator, and the dynamics within the solution [82]. A reversibility experiment was used to investigate the pH sensor response time. The dual-layer pH sensor was held in ASW and with the LED on. The ASW pH was repeatedly increased and decreased between pH_T_ 8.0 and 9.2 using 0.2 M HCl and 0.2 M NaOH for 65 min. The wavelength where the pH sensor was insensitive to pH change (320–420 nm) was normalized, and the wavelengths where iminocoumarin was most sensitive to pH change (535–545 nm) were plotted against time. The ResponseCurveFit.m MATLAB algorithm [83] was used to derive sensor response time (*t*_90_, *t*_95_, *t*_99_).

#### 2.5.4. Determination of Apparent p*K*_a_’ and LOD of the Dual-Layer pH Sensor via Inverse Calibration

The relationship between pH and *R* can be described using the modified Henderson–Hasselbalch equation:

(3) $${\mathrm{pH}}_{T}=\beta \times \log\frac{{(R}_{\mathrm{pH}}\;-\;R_{\mathrm{HIn}})}{{(R}_{\mathrm{Ind}-}\;-\;R_{\mathrm{pH}})}+{pK}_{a}^{’}$$

Data were collected through pH titration of ASW. This was conducted in a water-jacketed chamber with temperature monitored with a K-type thermocouple (±0.1 °C, NI-USB-TC01, National Instruments, Austin, TX, USA). The pH reference was of a non-refillable pH electrode (ECFC7252201B, Eutech Instruments, Singapore) connected to a high-input impedance electrode interface (EMF2, Lawson Labs, Malvern, PA, USA).

The pH of ASW was initially reduced to pH 6 with the addition of 0.2 M HCl. Then, the pH was increased in small steps (approximately 0.1–0.2 pH units) with 0.2 M NaOH to pH >10. The intensity change was found to be minimal when iminocoumarin was in its protonated (pH_HIn_ < 7.0) or deprotonated (pH_Ind-_ > 9.7) form. After each NaOH addition, five minutes were allowed for equilibration, and then *R* was calculated using the method described in Section 2.4.1. This allowed the estimation of p*K*_a_’ as the intercept from a linear regression, provided standard regression assumptions were met in the data.

**Figure 3:**
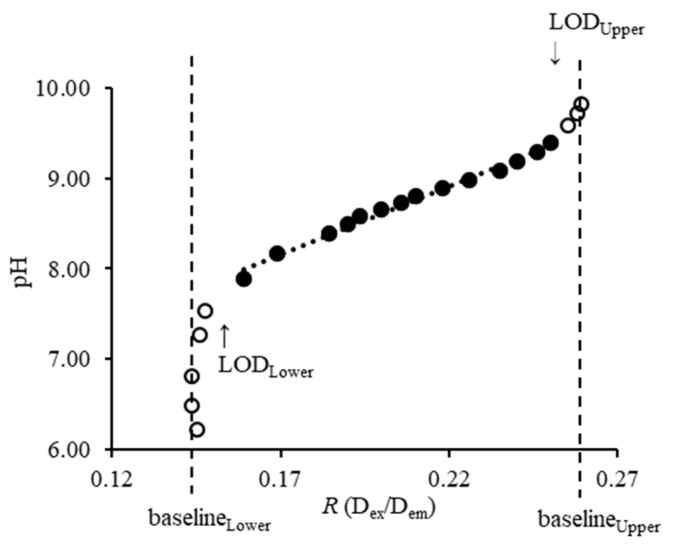
The approximately linear range of the pH sensor (●) was identified using LOD on the *R* scale, while values in the asymptotes (○) were averaged as baseline_Lower_ and baseline_Upper_ and used to establish LOD.

To accomplish this, it is important to consider broader concepts from linear and nonlinear calibration. First, we note that Equation (3) can be rewritten, where *R*_pH_ is a nonlinear function of pH_T_. Specifically, the response of this pH sensor is a sigmoidal curve, where pH is correctly treated as the independent variable and *R* as the response variable. The equation has lower and upper asymptotes, creating a range of pH values that cannot be reliably distinguished from each other (Figure 3).

One approach to modeling these data would be through nonlinear regression, where uncertainty in pH would be estimated by inverting prediction intervals from the regression curve [84], an approach termed ‘classical’ calibration. Unfortunately, classical nonlinear calibration is numerically challenging for this equation due to parameter identifiability issues unless calibration data are carefully chosen, and a simpler approach is desired.

The alternative is to swap response and independent variables, an approach termed ‘inverse’ calibration. Here, that means treating pH as the response and *R* as the independent variable as in Equation (3), and then performing a linear regression. Inverse calibration in this setting allows the use of standard linear regression tools, vastly simplifying calculations and providing good numerical stability. In linear systems, both classical and inverse calibration are commonly used with relatively small differences between them [85,86]. However, inverse calibration can perform poorly in nonlinear systems due to the differences in variability on the inverted scale, and failures are particularly pronounced at asymptotes [87]. So, in order to use inverse calibration for this sensor, an approximately linear range must first be identified and analysis restricted to that range. The approach here was based on first finding the limit of discrimination (LOD) on the *R* scale and visually inspecting whether that range would also be suitable for defining the approximately linear range. Because there are both lower and upper asymptotes, this leads to both lower and upper LODs.

The LOD of the pH sensor on the *R* scale is defined as the [H_3_O^+^] in the analyte solution that can be reliably distinguished from the background level. Based on standard acceptable false positive and false negative rates of 0.05 [88], this leads to

LOD_Lower, *R*_ = baseline_Lower_ + 3.3 × *s*_b, Lower_ (4)

LOD_Upper, *R*_ = baseline_Upper_ − 3.3 × *s*_b, Upper_ (5)

where baseline_Lower_ and baseline_Upper_ are the averaged *R* values in the respective asymptotes, i.e., pH_protonated, Lower_ < 7.0 and pH_deprotonated, Upper_ > 9.7, respectively, while *s*_b, Lower_ and *s*_b, Upper_ are the standard deviations in the respective regions. Conveniently, these also produced values that were in the approximately linear range of the response (Figure 3), and so were also used to restrict the range of data for performing inverse calibration.

Once data were restricted to the approximately linear range, a regression was performed based on Equation (3). From this, p*K*_a_’ is estimated by the intercept, and LOD on the pH scale is estimated using the estimated intercept and the estimated slope by

(6) $${\mathrm{LOD}}_{\mathrm{Lower},\;\mathrm{pH}}=\beta \times \log\frac{{(\mathrm{LOD}}_{\mathrm{Lower},\;R}\;-\;R_{\mathrm{HIn}})}{{(R}_{\mathrm{Ind}-}\;-\;{\mathrm{LOD}}_{\mathrm{Lower},\;R})}+{pK}_{a}^{’}$$

(7) $${\mathrm{LOD}}_{\mathrm{Upper},\;\mathrm{pH}}=\beta \times \log\frac{{(\mathrm{LOD}}_{\mathrm{Upper},\;R}\;-\;R_{\mathrm{HIn}})}{{(R}_{\mathrm{Ind}-}\;-\;{\mathrm{LOD}}_{\mathrm{Upper},\;R})}+{pK}_{a}^{’}$$

The dynamic range of the optical fiber sensor was defined as the range from the limit of quantification, and p*K*_a_’ was determined from the repetition of pH titrations using eight pH sensors from three different batches with results presented as 95% confidence intervals (*N* = 8, 95% CI). The standard deviation (*s*) of pH is calculated after conversion to [H_3_O^+^], and p*K*_a_’ results are presented as $\bar{\mathrm{pH}}$ (pH_min_, pH_max_) (*s*, *N* = sample size).

#### 2.5.5. Investigation of Environmental Influences on the pH Sensor

For the investigation of temperature influence on the pH sensor, the temperature of ASW (*S* = 35) was varied between 10 and 25 °C with a 5 °C interval, reflecting the temperature range (T = 8 to 22 °C) normally encountered in New Zealand waters [89]. For the investigation of salinity influence on the pH sensor, ASW with salinity at 35.0, 32.5, 30.0, 27.5, and 25.0 was prepared (T = 20 °C). This range (*S* = 25 to 35) mirrors estuarine and coastal systems. The pH titration of each of the temperature and salinity conditions was repeated three times.

#### 2.5.6. Sensor Precision and Accuracy

This pH sensor is intended to be used for the measurement of pH changes at the seaweed–DBL interface. The average temperature of Dunedin, New Zealand, is <15 °C and the seawater pH is approximately 8 [90,91]. Thus, seven Tris buffers (approximately evenly spaced, pH_T_ 8.28 to 9.45) were prepared and used to derive sensor precision. The experiment was repeated three times with a total of nine pH measurements for each Tris buffer. The first replicates were used for sensor calibration, and the replicate measurements were analyzed through pooled standard deviation (*s_pooled_*) to report sensor precision. The difference between the mean pH ($\bar{\mathrm{pH}}$) from the pH sensor and the pH electrode was used to determine sensor accuracy.

#### 2.5.7. pH Measurement within the DBL of *Ulva* sp.

*Ulva* sp. was collected from Kuri Bush (4th December 2022, S 46° 2′ 0″, E 170° 14′ 0″), and seawater (filtered and UV sterilized) was collected from the Portobello Marine Laboratory (2nd and 11th December 2022, S 45° 56′ 56″, E 170° 19′ 51″), Dunedin, New Zealand. The DBL experiment was conducted in a temperature-controlled room (13 ± 1 °C) to simulate the in situ temperature (13–14 °C [90]). During the light-on periods, cool white fluorescent bulbs (18 W, Phillips, Amsterdam, Netherlands) provided photosynthetically active radiation of 32 ± 3 μmol quanta m^−2^ s^−1^ (4π quantum meter, Biospherical Instruments, San Diego, CA, USA).

Approximately 5.5 g wet *Ulva* sp. was attached to a 20 × 7 cm^2^ polypropylene mesh and held in 1.8 L of seawater (*S* = 32.2). The pH sensor was placed at the DBL interface of the *Ulva* sp., at approximately 2 mm, assuming that this distance encompassed the DBL thickness, which is approximately μm to cm [13]. The reference pH electrode was placed at the opposite end of the chamber (2 L beaker), approximately 16 cm away from the seaweed, representing the bulk environment. Prior to pH measurement, *Ulva* sp. in the seawater was undisturbed with alternate light:dark cycles (12:12 h) allowing the build-up of the DBL.

At the end of the undisturbed periods of 12 h and 3 days, respectively, two experiments were conducted over 7 (undisturbed—12 h) and 5 (undisturbed—3 days) hours to examine the influence of DBL build-up over time on pH variations due to metabolic activity changes. The experimental data were acquired every 6 min for 1.5 s with periods of light and dark. The experimental chamber was not isolated in a dark box and the PMT is highly sensitive to any present stray light; thus, data acquisition during the light period was carried out with surrounding lights switched off for a brief 1.5 s, and then the lights were switched on again. A pH sensor coated with a bare sol-gel matrix without luminophores was used to offset background noise. Calibration data were acquired immediately before and at the end of the experiment using 4 Tris buffers (approximately evenly spaced, pH_T_ 8.1 to 9.7). These data were used to apply a time-correlated linear regression [48] to account for sensor drift. The pH_T_ changes in the DBL interface were calculated using Equation (3), and calibration uncertainty based on the equation was presented as 95% CI.

## 3. Results

### 3.1. Photostability and Indicator Leaching Investigation of the Dual-Layer pH Sensor

**Figure 4:**
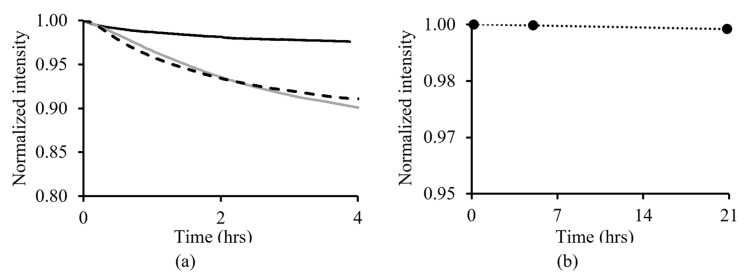
(**a**) Photostability of the optical sensor fabricated with iminocoumarin (⁃⁃⁃), Ru(dpp)_3_-PAN (▬), and dual-layer pH sensor (▬) in ASW (T = 20 °C). (**b**) Indicator leaching test results of the dual-layer pH sensor.

After being illuminated with the LED for 4 h, the optical intensity reduction due to photobleaching of iminocoumarin (4.86 × 10^−4^ min^−1^) and Ru(dpp)_3_-PAN (5.14 × 10^−4^ min^−1^) was approximately 10%. In comparison, the dual-layer pH sensor exhibited a greater photostability with an overall intensity reduction (1.16 × 10^−4^ min^−1^) of approximately 2%. In addition, the dual-layer pH sensor also had a higher overall intensity due to having heavier film thickness and higher dye concentration (Figure 4a). After immersing the sensor in ASW (pH_T_ approximately 8, T = 20 °C, *S* = 35) for 22 h, the intensity reduction due to indicator leaching was 0.14% (7.5 × 10^−5^ h^−1^) (Figure 4b).

### 3.2. Investigation of the Usable Lifetime of the pH Sensor

**Figure 5:**
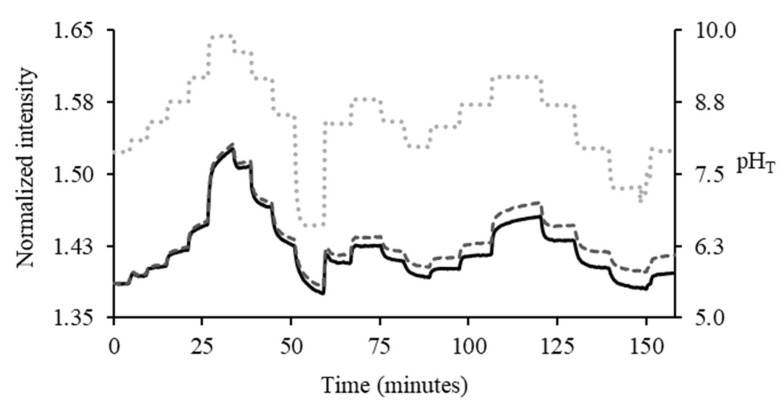
The pH sensor response to pH changes before (⁃⁃⁃) and after photobleaching drift adjustment (▬) over the 150 min experimental period. pH reading from an electrode (…) was used as a reference.

The normalized optical spectra of the pH sensor response to pH changes (Figure S13) showed three peak regions: the LED (460 to 480 nm), iminocoumarin (535 to 545 nm), and Ru(dpp)_3_ (600 to 620 nm). The wavelengths 535 to 545 nm, where iminocoumarin was most sensitive to pH change, were plotted against time. The determined photobleaching drift value (1.16 × 10^−4^ min^−1^) of the dual-indicator pH sensor from Section 3.1 was used to offset the optical intensity drift due to photobleaching. If the intensity drift due to photobleaching was not compensated for, it would lead to a −0.005 pH unit bias in the calculated pH value per minute. Thus, fluorescence intensity measurement is unreliable due to the degradation of the fluorophore. Figure 5 shows the pH sensor lifetime experiment over the 150 min experimental period with the pH sensor continuously exposed to the LED light.

### 3.3. Sensor Response Time

The pH sensor response to the pH change from pH 8.0 to 9.2 was more defined and faster than the change from pH 8.8 to 8.0 (Figure S14a). The first-order LTI model curve fitting [83] results showed the pH sensor had a response time (*t*_90_) of 22 s moving from pH 8.4 to 8.8 (Figure S14b), and 100 s moving from pH 8.8 to 8.4 (Figure S14c). In comparison, the pH electrode had a response time (*t*_90_) of 31 s (pH 8.4 → 8.8) and 28 s (pH 8.8 → 8.4) (Table 2).

### 3.4. Apparent pK_a_ and LOD of the Dual-Layer pH Sensor

**Figure 6:**
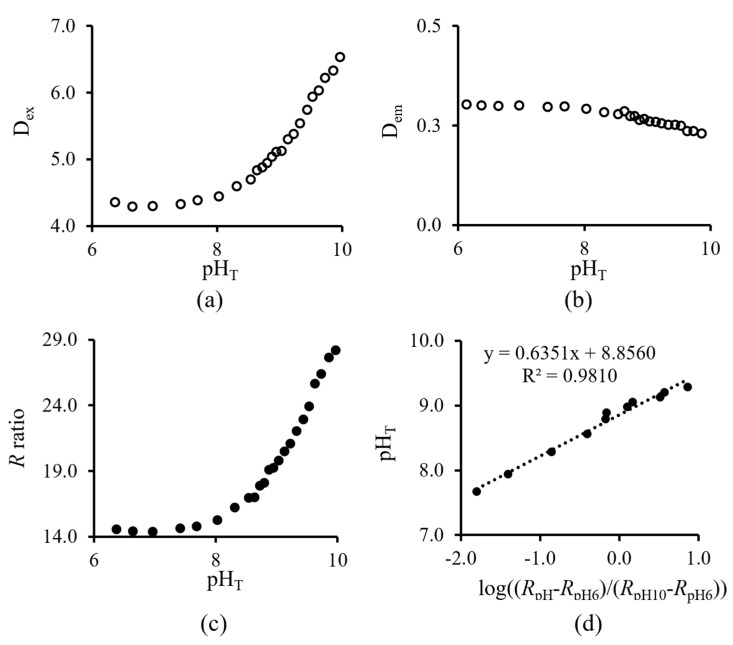
The area of (**a**) excitation (D_ex_), (**b**) emission (D_em_) intensity during the LED−on and LED−off period, and (**c**) the calculated *R* (i.e., pH response) is a sigmoidal shape. (**d**) The p*K*_a_’ of the pH sensor is 8.856 at 15 °C (S = 35).

Applying the background subtraction approach, the excitation area (D_ex_) of the pH sensor increased as the pH increased from pH_T_ 6 to 10 (Figure 6a), the pH-insensitive decay signal (D_em_) remained relatively constant (Figure 6b), and the calculated *R* had a sigmoidal pH response (Figure 6c). In the given example, at 15 °C (*S* = 35), the p*K*_a_’ of the pH sensor was determined as 8.86 (Figure 6d) by applying Equation (3).

Data processing using the *t*-DLR background subtraction approach is recommended if the experimental environment has substantial stray light interference. Further studies showed that if the experiment is conducted in a light-tight environment, the background noise correction could be omitted (Figure S15).

Evaluation of eight optical fiber pH sensors from three different batches found the p*K*_a_’ of the fluorescence pH sensor was determined as 8.65 (8.55, 8.75) (±0.10, *N* = 8, 95% CI) in ASW (T = 20 °C, *S* = 35), and the LOD_Lower_ and LOD_Upper_ of the pH sensors were 7.41 ± 0.20 and 9.42 ± 0.23 (*N* = 8, 95% CI), respectively.

### 3.5. Sensitivity to Temperature and Salinity

**Figure 7:**
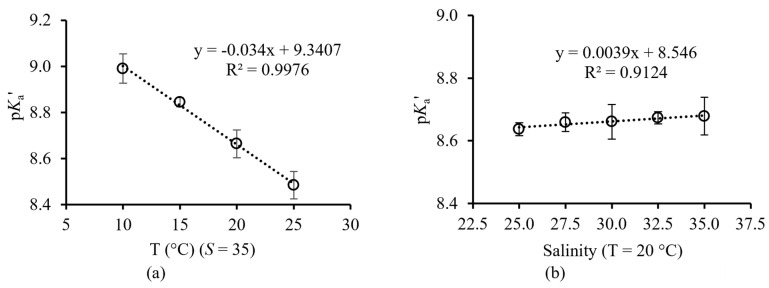
(**a**) The p*K*_a_’ of the pH sensor decreases with increasing temperature. (**b**) The pH sensor shows a negligible change in p*K*_a_’ with increasing salinity. Error bars denote the standard deviation of the associated p*K*_a_’.

The p*K*_a_’ of the optical fiber sensors decreased from 9.0 to 8.5 as the temperature increased from 10 to 25 °C (Figure 7a). The p*K*_a_’ of the optical fiber sensors increased from 8.64 to 8.68 as the salinity increased from 25 to 35 (Figure 7b).

### 3.6. Sensor Precision and Accuracy

The sensor precision, based on the pooled standard deviation of measurements in seven Tris buffers (pH_T_ 8.28 to 9.45, T = 10 °C), was 0.021 pH_T_ units. The pH sensor accuracy was 0.023 pH_T_ units, which was the difference between the mean pH reading of the dual-layer pH sensors and the pH electrode. The precision of the pH electrode used in the same experiment was 0.004 pH_T_ units (Table 3).

### 3.7. Real-Time pH Measurement of the Ulva sp. DBL Interface

Two experiments were performed with *Ulva* sp. that remained undisturbed for 12 h and 3 days to allow the build-up of metabolic gases and ions within the DBL. An optical fiber pH sensor was placed in the DBL adjacent to the *Ulva* sp. surface, whilst the pH electrode was placed at the opposite end of the chamber, mimicking bulk seawater.

**Figure 8:**
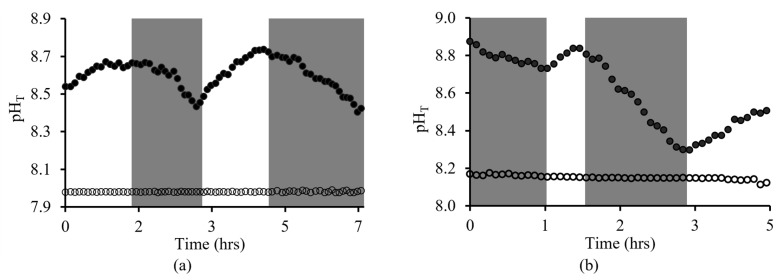
pH measurements with the dual-layer pH sensor (●) in the *Ulva* sp. DBL and the pH electrode (○) in bulk seawater after undisturbed periods of (**a**) 12 h and (**b**) 3 days, respectively. Shaded areas indicate periods of light off.

Following the 12 h undisturbed period, the pH at the DBL interface (~pH 8.5) was higher than the bulk seawater (~pH 8.0). The pH variation (8.4 to 8.7, ±0.3, 95% CI) reflected the expected pH increases during the light-on period due to photosynthesis and pH decreases during the light-off period due to respiration (Figure 8a). Following the 3-day undisturbed period, the pH at the DBL interface (~pH_T_ 8.7) was higher than the bulk environment (~pH_T_ 8.2), and larger differences in pH_T_ (8.3 to 8.9, ±0.2, 95% CI) (Figure 8b) were observed as compared to the 12 h undisturbed DBL. In both experiments, the pH reading of the pH electrode that represents the bulk environment remained relatively constant during light-on and light-off periods.

## 4. Discussion

The overall goal of this study was to measure the metabolic activities induced by pH changes in the DBL microenvironment, using the developed dual-layer fluorescent optical fiber pH sensors and the low-cost *t*-DLR interrogation system.

Photobleaching is one of the factors that cannot be compensated for using the *t*-DLR method. To counter the photobleaching effect, the pH-sensitive and pH-insensitive luminophore pairs used in the sensor fabrication should possess similarly high photostability. The dual-layer pH sensor had a greater photostability compared to sensors fabricated with a monolayer sol-gel matrix containing either iminocoumarin or Ru(dpp)_3_-PAN. This was due to the dual-layer sensor having a heavier film thickness (Figure S8) than that of the individual luminophore sol-gel coating, which led to a greater caging effect and further reduced the photodegradation of the luminophores. Results showed the intensity reduction due to photobleaching was similar for both of the monolayer sensors, implying that the preferential photobleaching of one of the luminophores is unlikely to occur. This finding confirms that the laboratory-synthesized iminocoumarin and Ru(dpp)_3_-PAN are well-suited for the *t*-DLR methodology.

The indicator leaching study of the dual-layer pH sensor showed a minor intensity drift (0.14% h^−1^) over the 22 h experimental period. This negligible indicator leaching should not impact the pH measurement using this *t*-DLR method. In addition, the optimized sol-gel matrix used in this work was tested previously in a DBL seawater condition continuously for 7 days, demonstrating its proven structural integrity in alkali corrosive seawater conditions [48]. Achieving a sensor with minimal leaching is possible through the covalent binding of the fluorophores to the sol-gel matrix. However, the introduction of an additional functional group to the indicator by covalent bonding could lead to the possibility of a p*K*_a_ shift outside the required pH range. Thus, caution must be taken in optical fiber pH sensor development, not only in the selection of suitable pH indicators but also in their immobilization methods, contemplating the appropriateness of the final sensor working range for the intended application.

Over the continuous 150 min exposure to the LED experimental period, the pH sensor was capable of determining pH changes. Once the photobleaching correction was applied, the stepwise pH changes remained clear and reproducible. These results indicated that the pH sensor has a minimum usable lifetime of 13 h using *t*-DLR interrogation at a frequency of 20 kHz (20% duty cycle) in a dark environment. Based on the findings, the pH sensor was shown to have the potential to monitor metabolic-induced pH changes in a marine microenvironment, such as diel (12 h on–off cycle) pH variations in seaweed DBL using the *t*-DLR pH measurement. However, if an intense background light source is present, e.g., daylight or cool white fluorescent bulbs, the usable lifetime of the pH sensor is expected to be shortened due to the increased risk of photobleaching.

The *t*-DLR interrogation system generated a lower pH reference signal compared to that of the pH-sensitive signal. This discrepancy arose from the LED’s 2 μs activation and 1 μs deactivation cycle, which restricted the usable time for the Ru(dpp)_3_-PAN (τ = 11 μs) signal to under 8 μs. To enhance the signal-to-noise ratio (SNR), it would be advantageous to explore alternative long-lifetime phosphorescent dyes, such as Pd(II) complexes, which can have a phosphorescence lifetime of up to 272 μs [92]. Utilizing such a long-lived luminophore could significantly augment the *t*-DLR’s integrated emission signal and improve SNR. However, when choosing a longer-lived luminophore, careful consideration is necessary to ensure that the regions in the excitation spectra for both the pH-sensitive fluorophore and pH-insensitive phosphorescence overlap for rationalizing the instrumentation setup.

The dynamic linear range of the fluorescent optical fiber pH sensor was 7.41 ± 0.20 and 9.42 ± 0.23. By taking the conservative values (i.e., 7.41+0.20 and 9.42–0.23), the working pH range was derived as approximately 7.6 to 9.2. These ranges are within the pH range of interest for pH measurements in marine environments (pH 7.5 to 9.5 [93]), indicating the pH sensor is suitable for the intended use. In addition, the response time (*t*_90_) of the dual-layer pH sensor (29 to 100 s) in this work was comparable with other fluorescent pH sensors developed for marine environment pH measurement applications (Table S4).

Temperature and salinity are key environmental influences that can affect the sensor performance and therefore must be compensated for. The influence of temperature on the pH sensor performance found that, if the change in p*K*_a_’ was not accounted for, it would lead to a 0.03 pH_T_ unit bias in the calculated pH_T_ value per degree (°C) increase in temperature. The pH sensor has negligible sensitivity in the salinity range of 25 to 35. The −0.004 pH_T_ unit bias in the calculated pH_T_ value increase per unit in salinity is within the standard deviation of the calculated p*K*_a_’. Thus, for field applications, compensation in temperature >1 °C is recommended, but salinity compensation is generally not needed.

The precision of the measurement was 0.02 pH_T_ (*s*), which is comparable to recognized fluorescent pH measurement techniques (Table S5). The sensor precision could be further improved through more consistent deposition of Ru(dpp)_3_-PAN onto the optical fibers during fabrication. However, the broad distribution size of Ru(dpp)_3_-PAN and the agglomeration nature has made it challenging to entrap a homogeneous Ru(dpp)_3_-PAN sol-gel coating onto the 400 μm fiber core. The precision of 0.02 pH_T_ does meet the “*weather*” quality guideline of GOA-ON which is intended to reflect sufficient precision for short-term biological and environmental studies.

The applicability of this sensor was demonstrated through real-time pH measurements of *Ulva* sp. in the discrete environment of the organism’s DBL. By monitoring pH fluctuations in the DBL, the optical fiber pH sensor detected metabolic activity directly attributed to photosynthesis and respiration. Inference from these experiments demonstrates the likely increase in DBL thickness due to metabolic activity buildup over time, shown by the increased pH fluctuation in the 3-day dormant DBL region (0.6 pH_T_ units), compared to that of the 12 h dormant DBL region (0.3 pH_T_ units). As expected, in both experiments, the initial pH measurement exhibited a higher pH in the DBL region than in the bulk environment, with a difference of approximately 0.6 to 0.7 pH_T_ units. The magnitude of this difference then increased or decreased, depending on metabolic activity.

The sol-gel matrix and pH-sensing layers employed in this study demonstrate remarkable versatility. These sensors have the potential for broader applications, including accommodating various indicators or even evolving into multi-analyte sensors. For example, integrating an O_2_-sensitive indicator into the sol-gel matrix could offer insights into metabolic processes within the DBL. Furthermore, the optical fiber sensor with CO_2_ and temperature-monitoring capabilities holds promise for applications in ventilation systems and horticulture. Expanding the pH sensor capabilities to include NO_2_ detection is applicable for agricultural and river outflow regions. In summary, this research successfully introduces an optical fiber fluorescent pH sensor for in situ measurements of the DBL, employing a DLR interrogation system. This work opens up a wide range of possibilities for environmental and industrial applications.

## 5. Conclusions

The optical fiber fluorescent pH sensor and the DLR interrogation system successfully measured changes in pH in the DBL of *Ulva* sp due to metabolic processes. This pH sensor has a p*K*_a_’ of 8.65 and a response time (*t*_90_) of 29 to 100 s (T = 20 °C, *S* = 35). This pH sensor and the supporting *t*-DLR instrumentation are suitable for marine environment pH measurements and could potentially contribute towards future studies on the impact of ocean acidification on marine calcifying species. The *t*-DLR instrumentation configuration produced pH data that conform to international quality guidelines with precision and accuracy of approximately 0.02 pH unit (*s*), which is comparable with previously reported fluorescence pH-sensing results. The pH sensor was insensitive to salinity, which is an appealing feature for pH sensors used for pH measurement in marine environments, where the salinity range often fluctuates in coastal and estuarine systems. This research highlights the practicability of optical fiber pH sensors by demonstrating real-time pH measurements of metabolically induced pH changes. The pH sensor and the lower-cost DLR instrumentation offer the potential to develop a field-deployable platform to monitor pH remotely and continuously in marine environments. This research highlights the versatility of the optical fiber pH sensors and the potential for a wider range of applications with the use of sol-gel-derived materials as the immobilizing agent.

## Figures and Tables

**Table 1:** Spectra and fluorescence properties of iminocoumarin, Ru(dpp)_3_, and Ru(dpp)_3_-PAN-SG (evaluated under ambient conditions).

		λ (nm)		τ (ns)			Φ	ε	Brightness
		ex	em	τ_1_	τ_2_	* χ^2^	(%)	(L mol^−1^ cm^−1^)	Φ × ε
Iminocoumarin	Ethanol	431	503	2.30		1.271	57.9	52,219	30,235
	pH 5.0	467	526	1.25	4.57	1.174	58.6	51,156	29,977
	pH 9.0	455	503	1.49	5.04	1.263	80.9	43,281	35,014
Ru(dpp)_3_	Ethanol	476	612				6.17	30,589	1887
Ru(dpp)_3_-PAN-SG	pH 8.2	468	620	11.66		1.104	40.3		

* Chi-square: quality of fit.

**Table 2:** The pH sensor response time (*t*_90,_ *t*_95,_ *t*_99_) was determined using the first-order LTI model curve fitting and drift was determined from the steady-state regions.

pH_T_ Changes	Types	*t*_90_	*t*_95_ (Sec)	*t*_99_	Drift (Intensity/Min)	The Goodness of Fit (*R^2^*)
8.4→8.8	pH electrode	31	36	48	2.0 × 10^−5^	1.0000
	pH sensor	29	34	44	1.5 × 10^−4^	0.9964
8.8→8.4	pH electrode	28	36	56	2.9 × 10^−3^	0.9969
	pH sensor	100	129	196	3.3 × 10^−4^	0.9987

**Table 3:** Reproducibility results of the pH sensor. Results are presented as mean $\bar{\mathrm{pH}}$ (pH_min_, pH_max_) and standard deviation (*s*).

pH Sensor					pH Electrode				
($\bar{\mathrm{pH}}$)	(pH_min_	pH_max_)	*s*	*N*	($\bar{\mathrm{pH}}$)	(pH_min_	pH_max_)	*s*	*N*
8.27	8.25	8.29	0.017	8	8.28	8.28	8.28	0.001	8
8.42	8.41	8.44	0.016	9	8.42	8.41	8.43	0.008	9
8.73	8.71	8.76	0.025	9	8.89	8.89	8.90	0.003	9
9.00	8.97	9.03	0.028	9	8.97	8.97	8.98	0.006	9
9.16	9.14	9.18	0.024	9	9.15	9.15	9.15	0.003	9
9.29	9.28	9.30	0.011	9	9.28	9.28	9.29	0.003	9
9.45	9.42	9.47	0.023	8	9.46	9.45	9.46	0.004	8
Precision (*s*_pooled_)			0.021	7				0.005	7
Accuracy			0.023	7				Reference	

## Data Availability

Not applicable.

## Supplementary Materials

The following supporting information can be downloaded at: https://www.mdpi.com/article/10.3390/s23218865/s1, Figure S1: HR-MS of iminocoumarin; Figure S2: ^1^H-NMR of iminocoumarin; Figure S3: ^13^C-NMR of iminocoumarin; Figure S4: ^1^H-NMR of Ru(dpp)_3_-PAN; Figure S5: DLS analysis of Ru(dpp)_3_-PAN; Figure S6: Iminocoumarin response to pH changes; Figure S7: Dual-luminophores response to pH changes; Figure S8: Microscopic views of sol-gel coated optical fibers; Figure S9: *t*-DLR instrumentation; Figure S10: LED driver circuit diagram; Figure S11: 2-stage PMT amplifier circuit diagram; Figure S12: Signal processing; Figure S13: Sensor usable lifetime; Figure S14: Sensor response time; Figure S15: Signal processing without background subtraction; Table S1: Sensor cost; Table S2: Comparison of iminocoumarin with other fluorescent dyes; Table S3: Sol-gel recipes of Ru(dpp)_3_-PAN and iminocoumarin; Table S4: Response time of fluorescence-based pH sensors; Table S5: Comparison of precisions of optical pH sensors. See Refs. [39,40,53,55,66,80,81,83,94,95,96,97,98,99].
