# Supplementary material for: Dual-Lifetime Referencing (t-DLR) Optical Fiber Fluorescent pH Sensor for Microenvironments
Source: Sensors (Basel). 2023 Oct 31;23(21):8865. doi: 10.3390/s23218865 (PMC10650387; doi:10.3390/s23218865)
Supplement: Supplementary file 1 [file sensors-23-08865-s001.zip › sensors-2641765-supplementary.pdf]

Contents

Sensor cost 1
Spectra and structure characterization of iminocoumarin 3
Dynamic light scattering (DLS) characterization of $Ru(dpp)_3$-PAN particles 6
t-DLR interrogation system 8
Sensor usable lifetime 10
Sensor response time 11
Signal processing without background subtraction 12

Sensor cost

Table S1. The cost of the main interrogation components typically used in DLR. The total cost for the *t*-DLR pH sensor in this work was approximately \$3,400 USD.

Methods	Typical electronic requirement	Cost (USD)
f-DLR [1, 2]	Dual-phase lock-in amplifier	> \$ 6000
t-DLR [3, 4]	Pulse generator	> \$ 4000
	Photon counting device and DAC system	> \$ 4000 to \$ 8000
This work:<br>*t*-DLR system	 Electronic components<br>(Optical components)	 \$ 1300<br>(\$ 2100)

Table S2. Comparison of iminocoumarin with other commonly used optical sensing fluorescent dyes with a pH-sensitive range between 6 to 8.

**Dye**	**pK_a (pH range)**	**Measurement Wavelengths**	**Application Notes**	**Features**	**Price (NZD)**
SNARF [1]	~7.5<br>6.0–8.0	λ_{ex} = 514 or 488 nm<br>λ_{em} = 580/640 nm	Ideal conjugate for ratiometric emission measurements.	Low quantum yield	$885 (1 mg)
BCECF [2]	~7.0<br>6.5–7.5	λ_{ex} = 490/440 nm<br>λ_{em} = 530 nm	Ideal conjugate for ratiometric excitation measurements.	Poor photostability	$489 (1 mg)
CF [3]	6.5<br>6.0–8.0	λ_{ex} = 470 nm<br>λ_{em} = 492 nm<br>λ_{em} = 514 nm	High quantum yield in alkali	Hydrophilic, low Stokes shifts, sensitive to IS	$101 (1 g)
HPTS [4, 5]	5.4, 7.4<br>(6.0–8.0)	λ_{ex} = 465 nm,<br>λ_{ex} = 450/405 nm<br>λ_{em} = 510 nm	High quantum yield	Hydrophilic, sensitive to IS	$244 (1 g)
Imino-coumarin [6]	8.05<br>(5.0-10.0)	(pH 2)<br>λ_{ex} = 480, λ_{em}= 520<br>(pH 11)<br>λ_{ex} = 430, λ_{em}= 480	High quantum yield	Insensitive to IS	Laboratory synthesis

CF - 5(6)-Carboxyfluorescein, HPTS - 8-Hydroxypyrene-1,3,6-trisulfonic acid trisodium salt, BCECF - 2′,7′-Bis(2-carboxyethyl)-5(6)-carboxyfluorescein, SNARF – seminaphthorhodafluor, SNAFL – seminaphthorfluorescein

Spectra and structure characterization of iminocoumarin

The melting point of the synthesized iminocoumarin was 239 ± 1.3 °C (literature: 238 °C [7]).

#	Score	Pred. (M)	Pred. m/z	Meas. m/z	Diff. (mDa)	Formulae (M)	Ion	Diff. (ppm)	Iso Score	DBE
1	56.02	332.16371	333.17099	333.17136	0.37	C20 H20 N4 O	[M+H]+	1.111	51.71	13.0

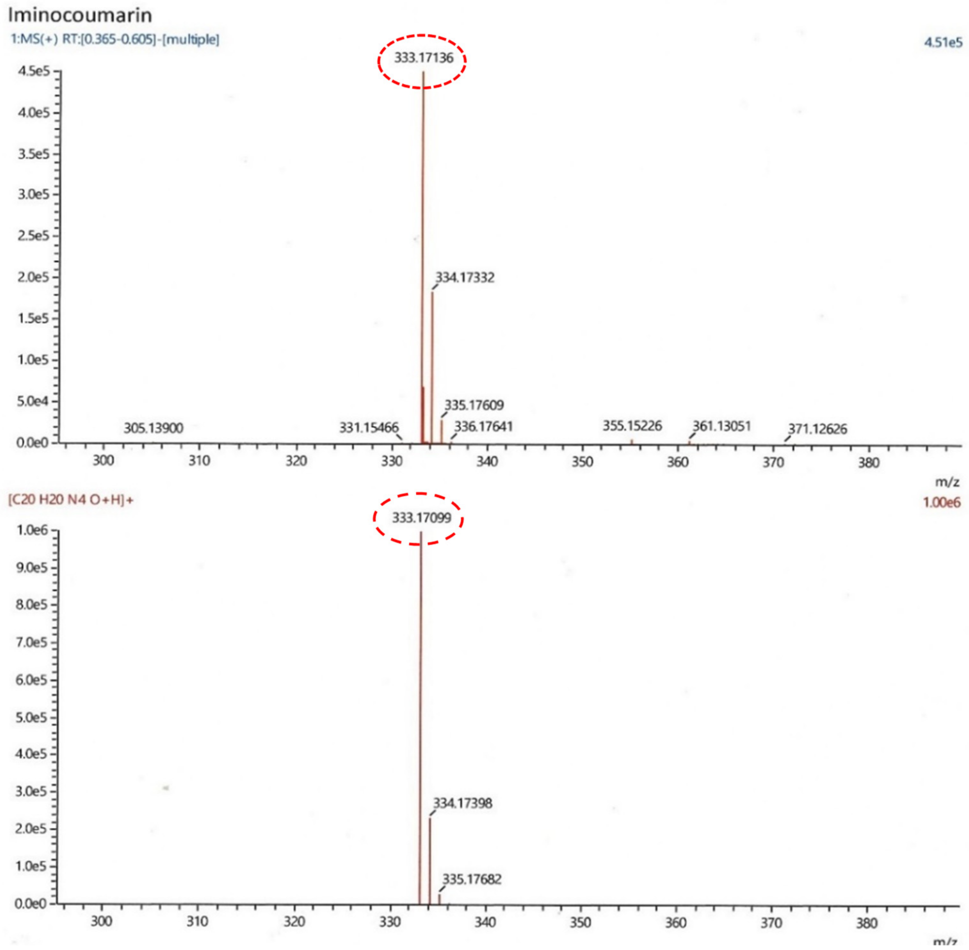

Figure S1. HR-MS spectrum of the synthesized iminocoumarin. Measurements were conducted by the Technical Support Team from the Chemistry Department of the University of Otago.

[1]**H NMR** (500 MHz, TFA-d) δ 11.50 (s, 3H), 8.82 (dd, *J* = 107.5, 5.3 Hz, 1H), 8.04 (d, *J* = 8.7 Hz, 1H), 7.80 (dt, *J* = 6.7, 3.3 Hz, 2H), 7.74 – 7.65 (m, 2H), 7.62 – 7.55 (m, 2H), 3.77 (q, *J* = 7.2 Hz, 4H), 1.28 (t, *J* = 7.2 Hz, 6H).

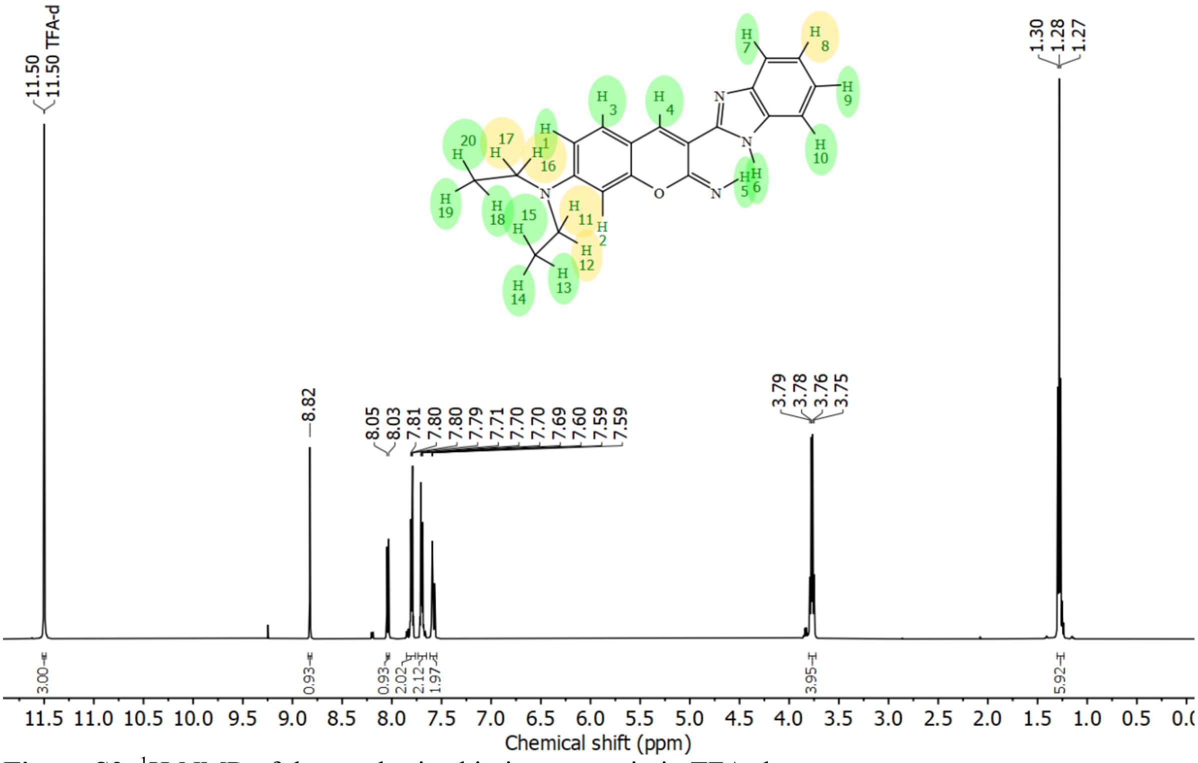

Figure S2. ^{1}H-NMR of the synthesized iminocoumarin in TFA-d.

[13]**C-NMR** (101 MHz, TFA-d) δ 167.78, 154.57, 152.82, 143.26, 137.60, 134.53, 132.59, 122.99, 119.92, 118.89, 117.64, 110.23, 55.68, 13.23.

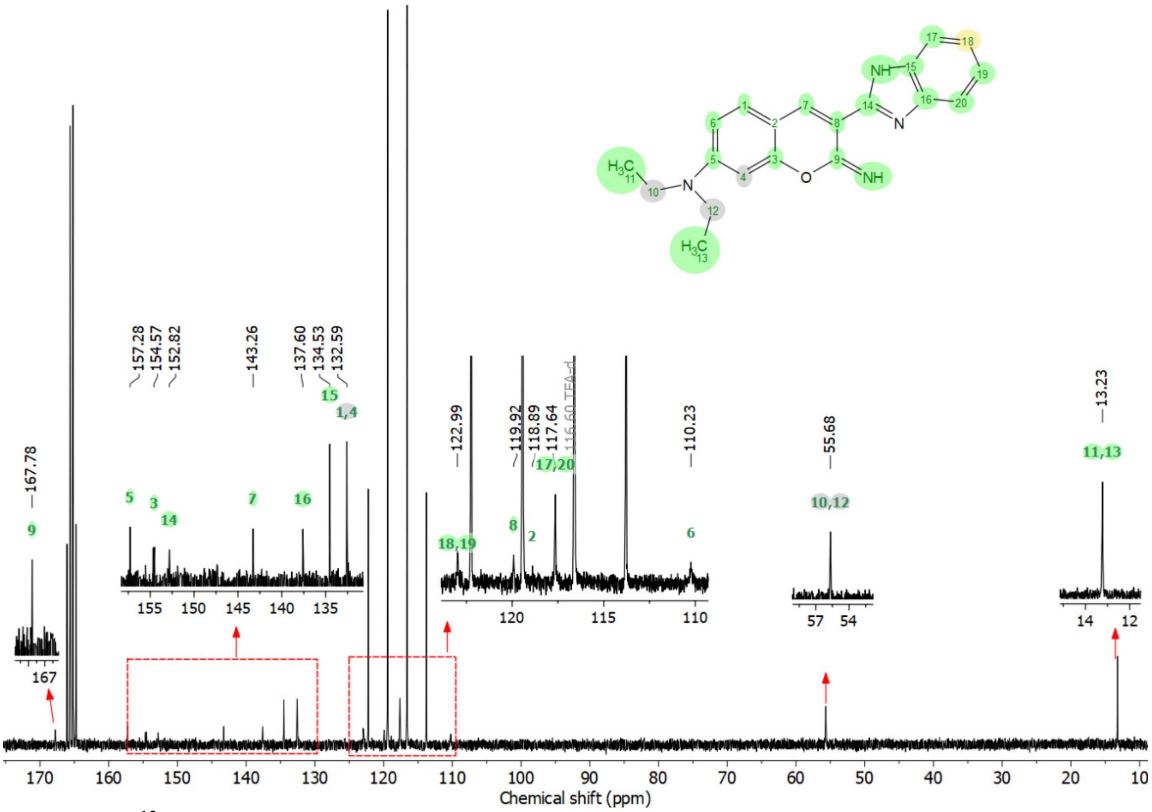

Figure S3. ^{13}C-NMR of the synthesized iminocoumarin in TFA-d.

^{1}H-NMR of the fabricated Ru(dpp)$_3$-PAN particles

The peaks marked 1 and 2 are αH and βH proton peaks of PAN, respectively. The peaks marked 3 between chemical shifts, 8.5 to 7.6 ppm are proton peaks of a Ru(dpp)$_3$ molecule (proton integration H=48). Proton integration ratio of Ru(dpp)$_3$ to PAN (α : β) are 0.02 : (1.00 : 0.50). The ^{1}H NMR of Ru(dpp)$_3$-PAN showed no DMF peaks [8] at 7.95, 2.89, and 2.75 ppm (**Figure S4**).

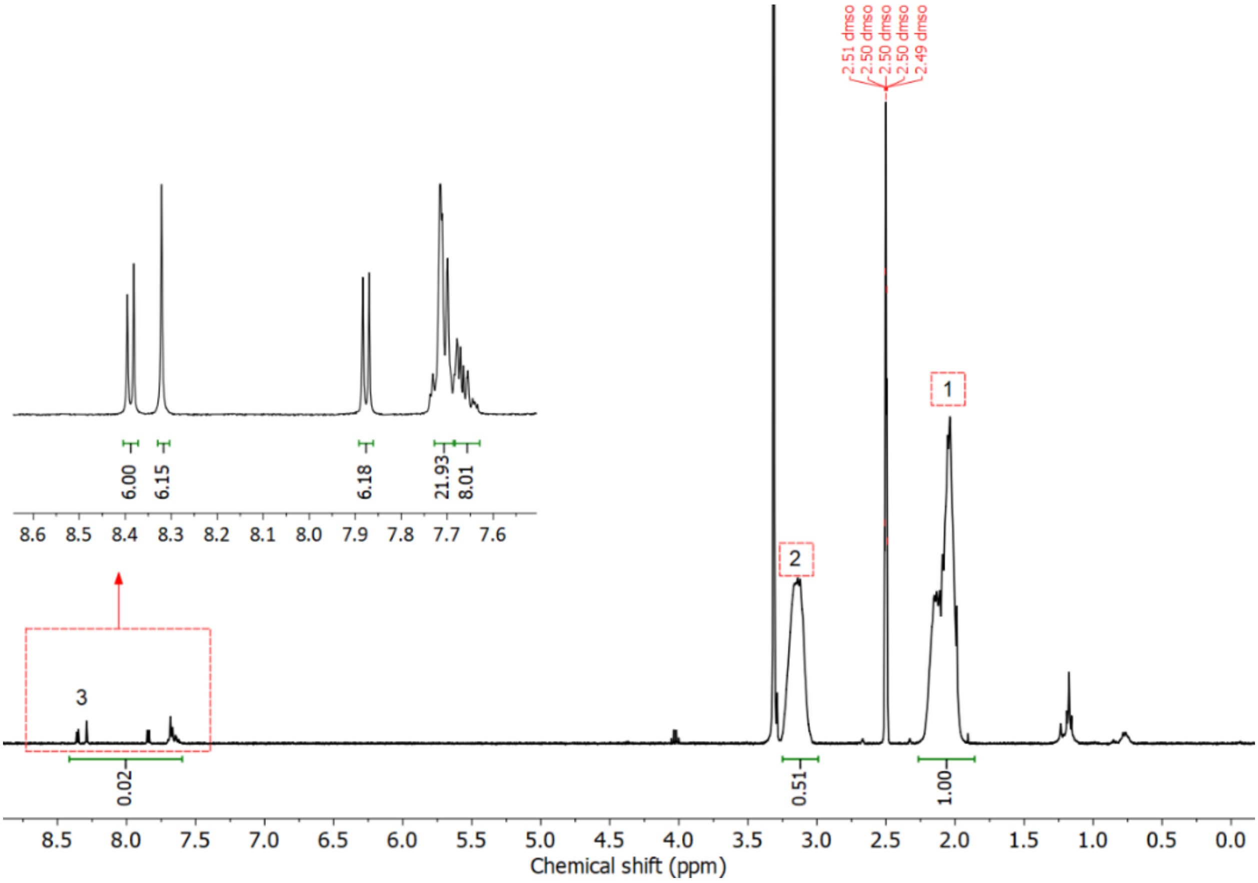

Figure S4. ^{1}H-NMR (400 MHz) spectrum of Ru(dpp)$_3$-PAN particles dissolved in DMSO-d_6.

Dynamic light scattering (DLS) characterization of $Ru(dpp)_3$-PAN particles

The polydispersity index (PDI) of the $Ru(dpp)_3$-PAN particles was found to be 0.513, which is within the range of 0.05 and 0.7 where DLS analysis is considered suitable for measuring the particle size of suspensions. However, the DLS measurement revealed a broad size distribution of $Ru(dpp)_3$-PAN, suggesting the presence of aggregation in the samples as shown by two peaks in the size distribution results (Figure S5a, Figure S5b).

The zeta potential of the synthesized $Ru(dpp)_3$-PAN particles was found to be negative 5.52 ± 0.1 mV. Zeta potential in the range of 0 to 5 mV was described as rapid coagulation or flocculation, and 40 to 60 mV is regarded as having good stability [9]. Thus, the synthesized $Ru(dpp)_3$-PAN with a zeta potential value of 5.52 ± 0.1 mV will likely lead to agglomeration. The synthesized $Ru(dpp)_3$-PAN particles are probably composed of individual nanospheres (< 60 nm) held together by electrostatic attraction in the form of agglomerates.

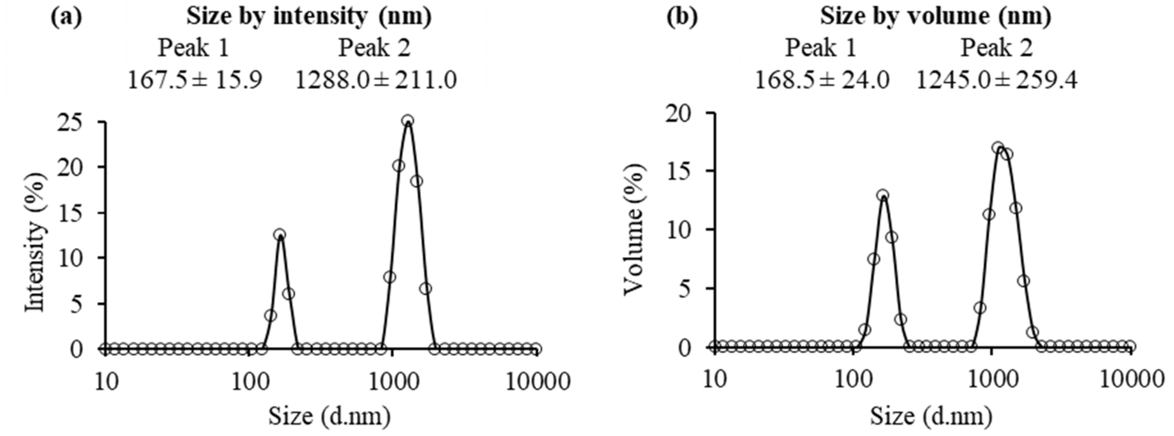

Figure S5. $Ru(dpp)_3$-PAN size distribution results using DLS measurement are presented as the average and standard deviation by the (a) intensity and (b) volume.

Fluorescence spectra

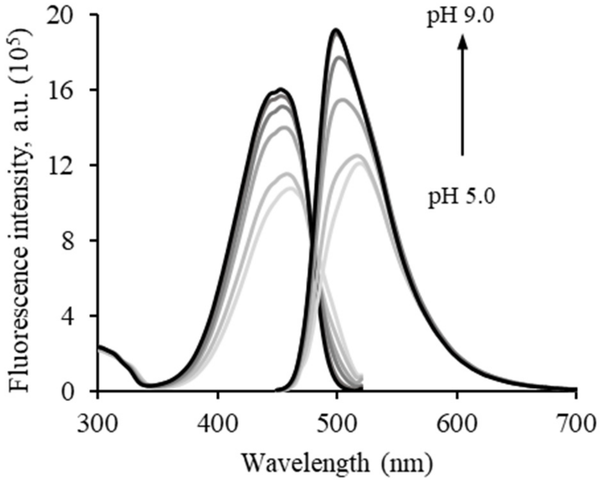

Figure S6. Fluorescence spectra of iminocoumarin showed the indicator was pH-dependent. In a range of pH buffer solutions, the excitation and emission fluorescence intensity of iminocoumarin increased as pH increased from 5 to 9 (light to dark).

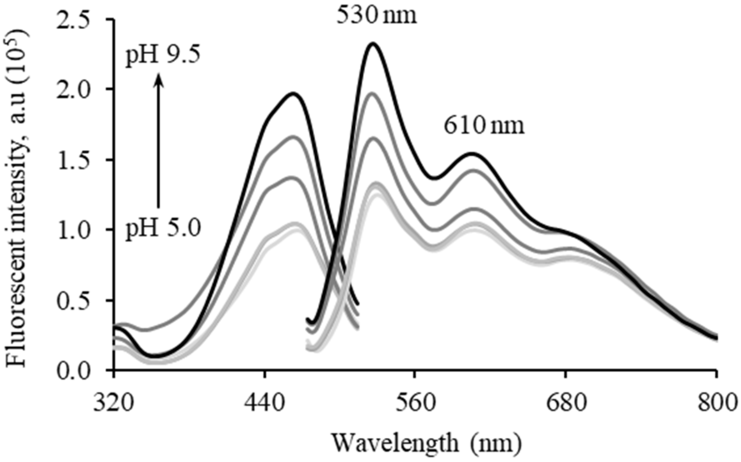

Figure S7. Dual-luminophores sol-gel coated glass slide in buffer solutions from pH 5 to 9.5 (IS = 0.7 M) showed emission peaks at 530 and 610 nm and broad absorbance of 380 to 480 nm. The fluorescence intensity of the dual luminophores increased as pH increased from pH 5 to 9.5 (light to dark).

Table S3. Ru(dpp)$_3$-PAN and iminocoumarin sol-gel blend. The dual-layer pH sensor comprised two sol-gel coatings, an inner layer of Ru(dpp)$_3$-PAN (active Ru(dpp)$_3$ 0.35 mM), and an outer layer of iminocoumarin (5.3 mM).

			Ru(dpp)$_3$-PAN sol-gel				Iminocoumarin sol-gel			
	MW (g mol^{-1})	Density (g mL^{-1})	(mL)	(gram)	(mol)	molar ratio	(mL)	(gram)	(mol)	molar ratio
Precursor						(1.00)				(1.00)
TEOS	208.33	0.933	1.850		8.29×10^{-3}	0.80	1.000		4.48×10^{-3}	0.79
DDS	148.28	0.865	0.360		2.10×10^{-3}	0.20	0.200		1.17×10^{-3}	0.21
Ethanol	46.07	0.789	1.280		2.19×10^{-2}	2.11	1.090		1.87×10^{-2}	3.31
Iminocoumarin	332.17							4.80×10^{-3}	1.44×10^{-5}	2.60×10^{-3}
Ru(dpp)$_3$-PAN				9.00×10^{-2}						
(Ru(dpp)$_3$)	1169.17			(1.76×10^{-3})	(1.51×10^{-6})	(1.45×10^{-4})				
Triton X-100	647	1.07		6.10×10^{-2}	9.43×10^{-5}	9.08×10^{-3}		3.35×10^{-2}	5.18×10^{-5}	9.20×10^{-3}
0.1 M HCl	36.46		0.300		3.00×10^{-5}	2.89×10^{-3}	0.165		1.65×10^{-5}	2.90×10^{-3}
Milli-Q	18	1	0.455	0.752	4.18×10^{-2}	4.03	0.245	0.409	2.27×10^{-2}	4.02
		TOTAL (mL)	~ 4.3				~ 2.7			

	Optical fibres with sol-gel coatings	Entrapped luminophores	Film thickness
(a)	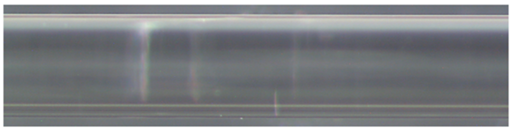	400 μm optical fibre (bare)	NA
(b)	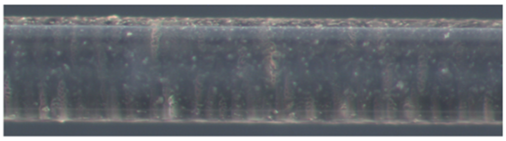	Ru(dpp)$_3$-PAN sol-gel coating	5.3μm
(c)	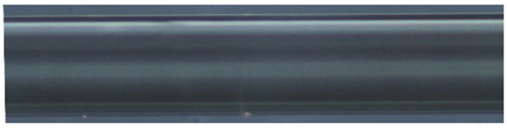	Iminocoumarin sol-gel coating	1.3 μm
(d)	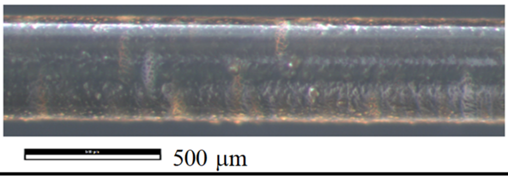 500 μm	Two layers of sol-gel coatings: 1st layer Ru(dpp)$_3$-PAN 2nd layer iminocoumarin	6.6 μm

Figure S8. The microscope views (obtained with Leica DFC295) of (a) a bare fiber without coating, (b) Ru(dpp)$_3$-PAN sol-gel coating (dip-coating speed: 58 cm min^{-1}), (c) iminocoumarin sol-gel coating (dip-coating speed: 25 cm min^{-1}), and (d) the dual-layer pH sensor with two layers of sol-gel coatings.

t-DLR interrogation system

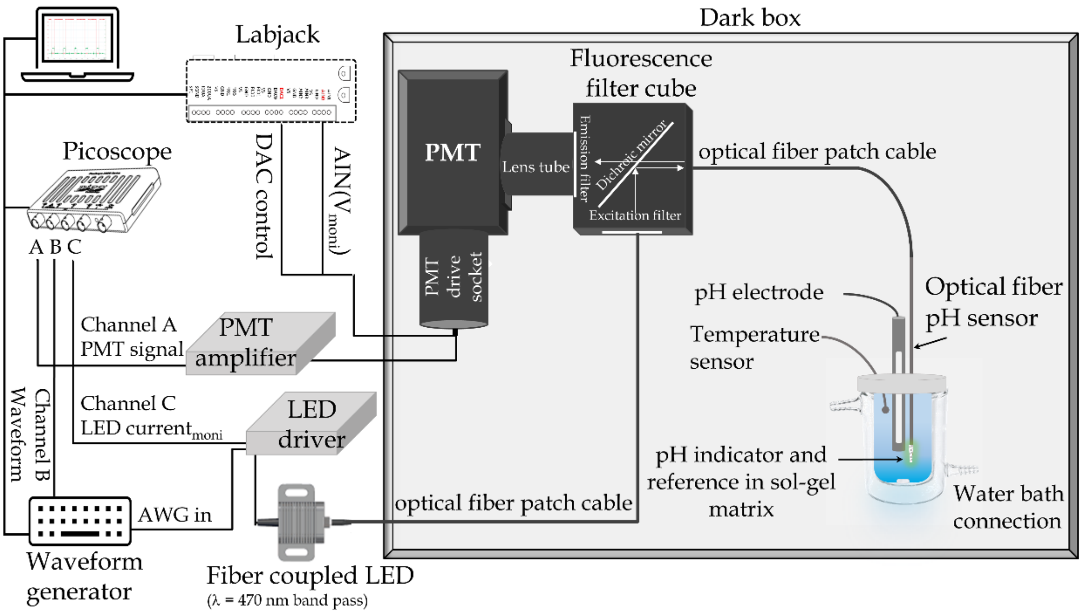

Figure S9. The *t*-DLR instrumentation.

LED driver

The frequency and time for opening and closing the current diversion were dictated by the frequency and duty cycle of the AWG. In this case, the set duty cycle was referred to as the completion of an off-on cycle of the current diversion, which was an inverted on-off cycle with respect to the LED, e.g., with a frequency of 20 kHz and a duty cycle of 80%, the LED was on for 10 µs and off for 40 µs.

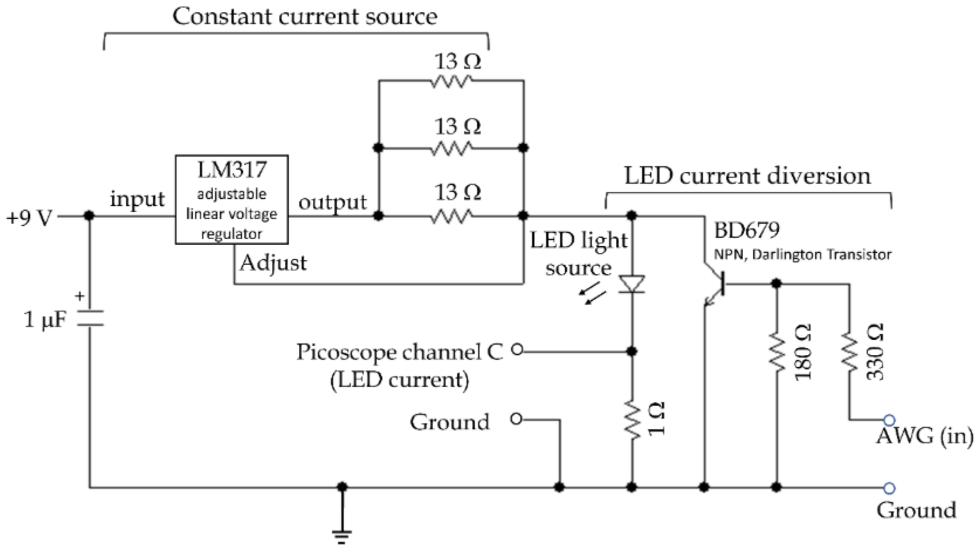

Figure S10. The LED driver circuit diagram (designed by Mr. Paul Reynolds).

The 2-stage PMT amplifier

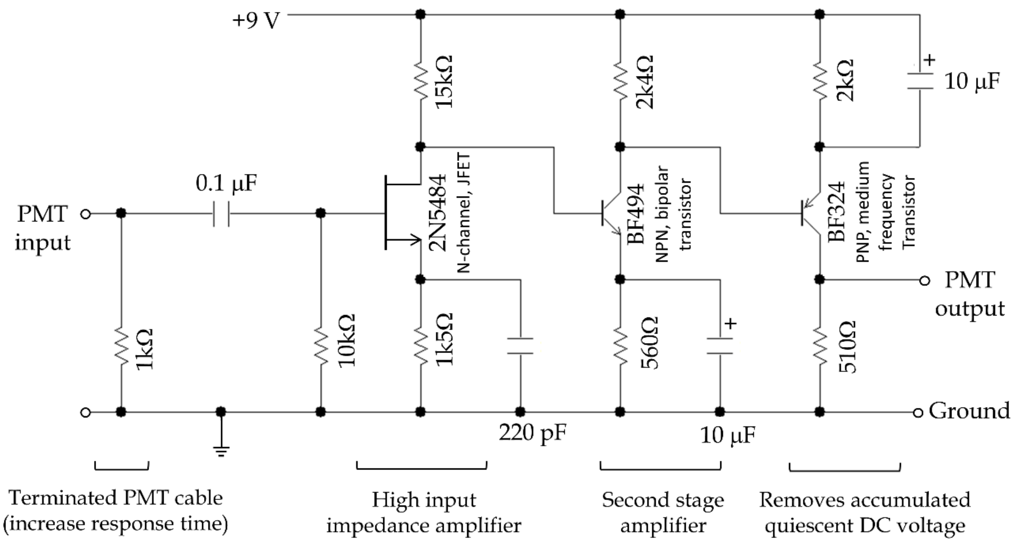

Figure S11. The 2-stage PMT amplification circuit diagram (designed by Mr. Paul Reynolds).

(a)

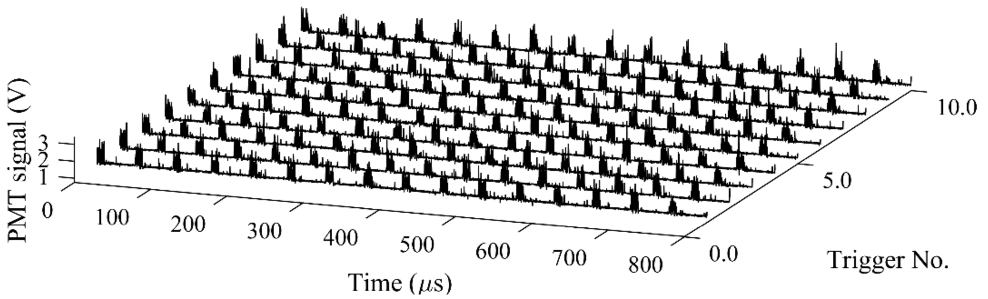

(b)

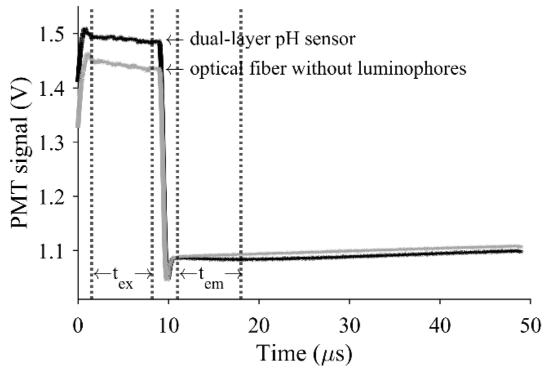

(c)

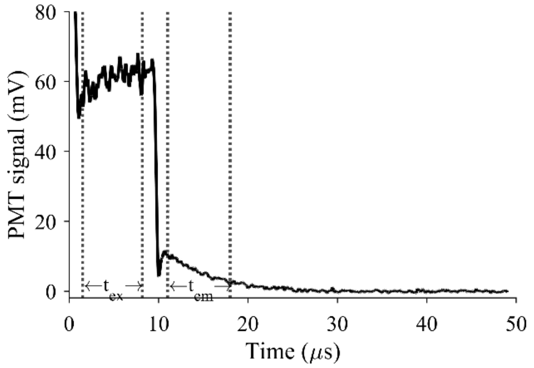

Figure S12. (a) A snapshot of the acquired signal (30,000 pulses). **(b)** A single on-off profile of the dual-layer pH sensor (▬) was obtained by averaging the 30,000 on-off cycles. The signal of an optical fiber without luminophores (▬) was plotted for comparison. The periods: t_{ex} and t_{em}, used to obtain excitation and emission intensity integration were denoted (**…**). **(c)** Signal appearance after background subtraction.

Sensor usable lifetime

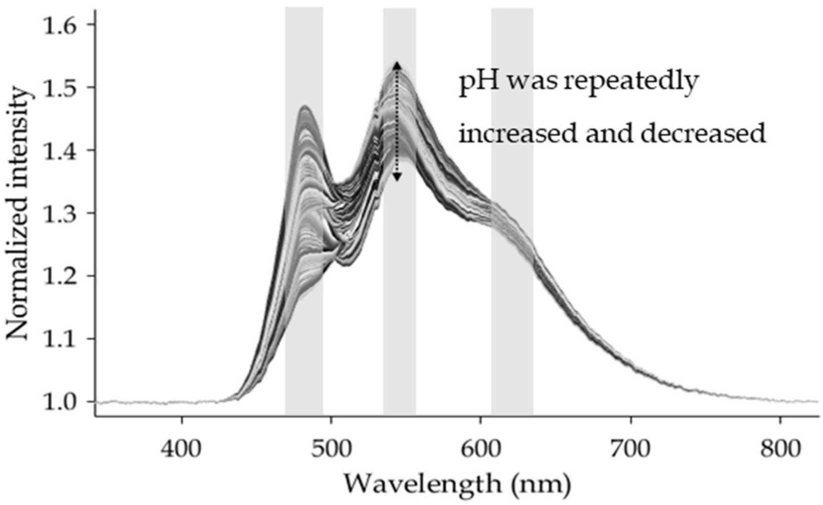

Figure S13. The normalized optical spectra of the pH sensor response to pH changes under continual exposure to the LED for 150 minutes. Highlighted areas indicate the LED (460 to 480 nm), iminocoumarin (535 to 545 nm), and $Ru(dpp)_3$ (600 to 620 nm).

Sensor response time

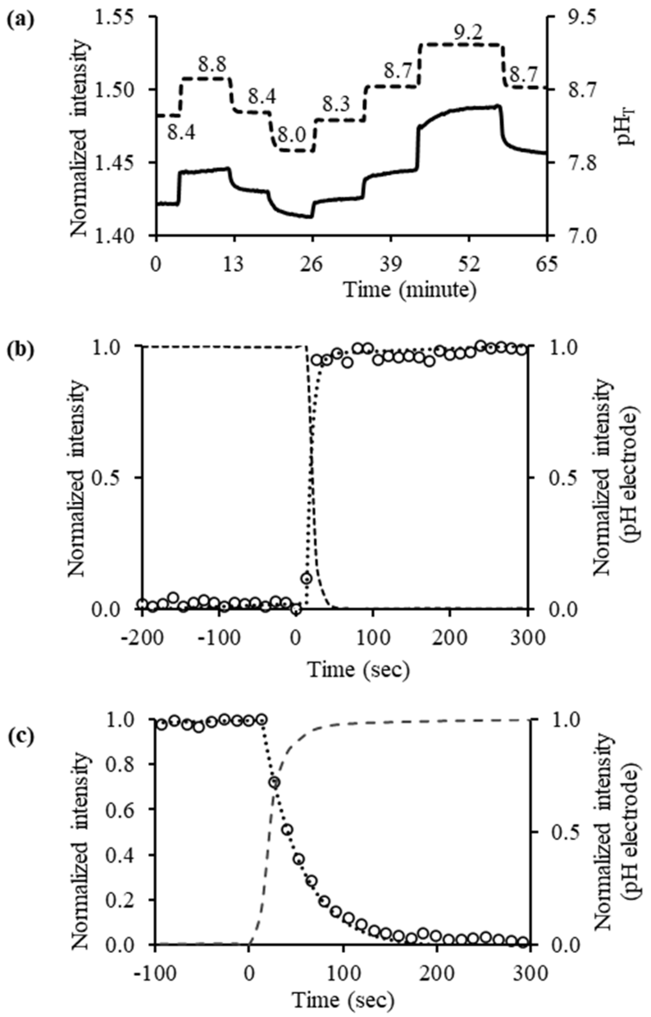

Figure S14. **(a)** The fluorescence optical fiber pH sensor response to pH change in ASW (*S* = 35, T = 20 °C) (**—**). For reference, pH$_T$ measured with a pH electrode (**---**) is shown. First-order LTI model curve fitting of the pH sensor (○) **(b)** from pH 8.4 to 8.8, and **(c)** from pH 8.8 to 8.4. The pH electrode (**---**) was used as a reference. Dotted lines (**…**) are the fitted curves using the MATLAB algorithm (time-response model “first-order LTI model” [10, 11]).

Table S4. Reported response time of fluorescence-based pH sensors.

Reference	Indicator	Matrix	Application	Response time	(s)
Clark, et al. [12]	pH sensor spot from PreSens	Unknown	pH optode for seawater pH measurement	(t_{95}) pH 7.2 to 8.5	50
Larsen, et al. [13]	HPTS, Macrolex Yellow	Polyurethane hydrogel (Hydromed D4)	pH distribution in marine sediments	($t_?$) pH 6.5 to 8.9	60
Schröder, et al. [14]	DHFA<br>DHFAE	Polyurethane hydrogel	pH measurement in marine environments	(t_{90}) pH 8.5 to 7.5<br>(t_{90}) pH 7.5 to 8.5	120<br>230
Current work	Iminocoumarin<br>Ru(dpp)$_3$-PAN	Sol-gel matrix with TEOS and DDS	Optical fiber pH Sensor for marine environments	(t_{99}) pH 8.4 to 8.8<br>(t_{99}) pH 8.8 to 8.4	44<br>196
				(t_{95}) pH 8.4 to 8.8<br>(t_{95}) pH 8.8 to 8.4	34<br>129
				(t_{90}) pH 8.4 to 8.8<br>(t_{90}) pH 8.8 to 8.4	29<br>100

Signal processing without background subtraction

pH titration (pH 6.5 to 10) of ASW (S = 35) at 15 °C using the dual-layer pH sensor. Signals were acquired using a 20 kHz pulse frequency (LED-on for 10 µs, LED-off for 40 µs).

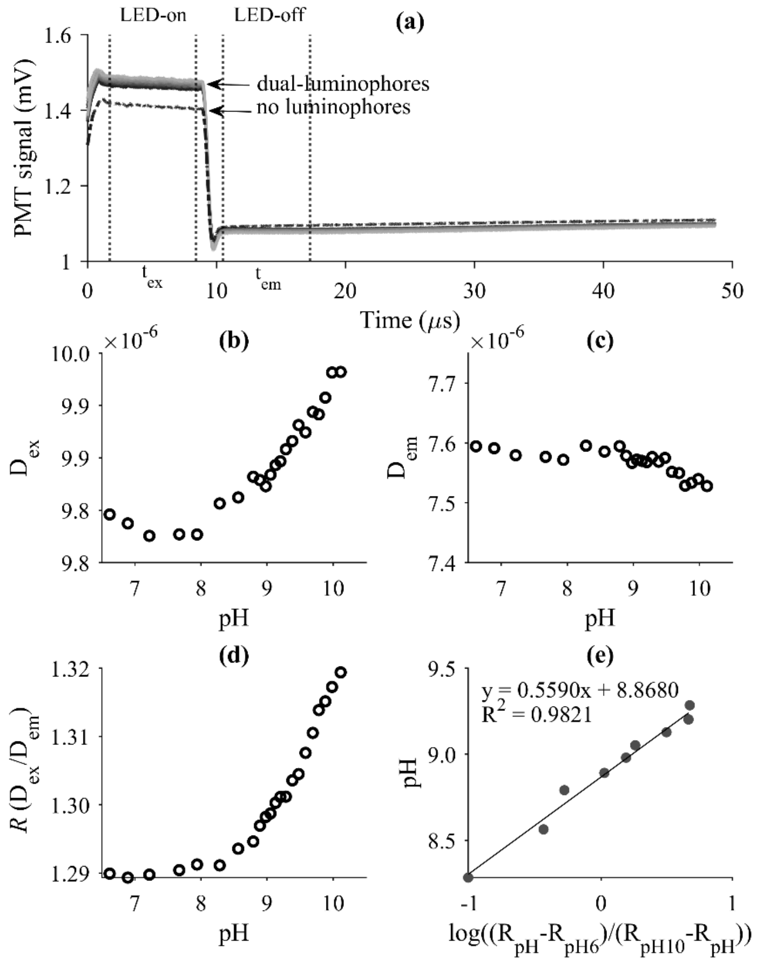

Figure S15. (a) The averaged signal of the dual-layer pH sensor and the background signal of an optical fiber without indicators (plotted as a comparison). The selected LED on and off regions are denoted as (**…**). Integration of signal intensity during **(b)** LED-on (D_{ex}), and **(c)** LED-off (D_{em}). **(d)** The calculated R values have a sigmoidal pH response curve. **(e)** The pK_a' of the pH sensor was determined as 8.87 (S = 35, T = 15 °C).

Sensor precision

Table S5. Reported optical pH sensors with similar precision to the sensor in this study.

Reference	Precision	Response time	Sensor materials	Technique
Schröder, et al. [14]	0.02	<200s	DHFA and DHFAE in hydrogel	*t*-DLR/CCD
Wencel, et al. [5]	0.02	12 sec	HPTS in sol-gel	Ratiometric
Larsen, et al. [13]	0.02	60 sec	HPTS and macrolex yellow coumarin in hydrogel	Ratiometric CCD
Current work	0.02	(t_{90}) 29 to 100 sec<br>(t_{95}) 34 to 129 sec<br>(t_{99}) 44 to 196 sec	Iminocoumarin / Ru(dpp)$_3$-PAN in sol-gel	*t*-DLR

References

1. Han, J.; Burgess, K. Fluorescent indicators for intracellular pH. *Chem Rev* **2010**, *110*, 5, 2709-28, https://doi.org/10.1021/cr900249z

2. Boens, N.; Qin, W.; Basaric, N.; Orte, A.; Talavera, E. M.; Alvarez-Pez, J. M. Photophysics of the fluorescent pH indicator BCECF. *J Phys Chem A* **2006**, *110*, 30, 9334-43, https://doi.org/10.1021/jp0615712

3. Liebsch, G.; Klimant, I.; Krause, C.; Wolfbeis, O. S. Fluorescent imaging of pH with optical sensors using time domain dual lifetime referencing. *Anal Chem* **2001**, *73*, 17, 4354-63, https://doi.org/10.1021/ac0100852

4. Chandra, A.; Prasad, S.; Iuele, H.; Colella, F.; Rizzo, R.; D'Amone, E.; Gigli, G.; Del Mercato, L. L. Highly Sensitive Fluorescent pH Microsensors Based on the Ratiometric Dye Pyranine Immobilized on Silica Microparticles. *Chemistry* **2021**, *27*, 53, 13318-13324, https://doi.org/doi.org/10.1002/chem.202101568

5. Wencel, D.; MacCraith, B. D.; McDonagh, C. High performance optical ratiometric sol–gel-based pH sensor. *Sensors and Actuators B: Chemical* **2009**, *139*, 1, 208-213, https://doi.org/10.1016/j.snb.2008.12.066

6. Vasylevska, A. S.; Karasyov, A. A.; Borisov, S. M.; Krause, C. Novel coumarin-based fluorescent pH indicators, probes and membranes covering a broad pH range. *Anal Bioanal Chem* **2007**, *387*, 6, 2131-41, https://doi.org/10.1007/s00216-006-1061-6

7. Chemate, S. B.; Sekar, N. Novel Iminocoumarin Derivatives: Synthesis, Spectroscopic and Computational Studies. *J Fluoresc* **2015**, *25*, 6, 1615-28, https://doi.org/10.1007/s10895-015-1648-4

8. Gottlieb, H. E.; Kotlyar, V.; Nudelman, A. NMR Chemical Shifts of Common Laboratory Solvents as Trace Impurities. *J Org Chem* **1997**, *62*, 21, 7512-7515, https://doi.org/10.1021/jo971176v

9. Kumar, A.; Dixit, C. K., 3 - Methods for characterization of nanoparticles. In *Advances in Nanomedicine for the Delivery of Therapeutic Nucleic Acids*, Nimesh, S.; Chandra, R.; Gupta, N., Eds. Woodhead Publishing: 2017; pp 43-58.

10. Frankaer, C. G.; Sorensen, T. J. A unified approach for investigating chemosensor properties - dynamic characteristics. *Analyst* **2019**, *144*, 7, 2208-2225, https://doi.org/10.1039/c9an00268e

11. Frankaer, C. G.; Sorensen, T. J. Investigating the Time Response of an Optical pH Sensor Based on a Polysiloxane-Polyethylene Glycol Composite Material Impregnated with a pH-Responsive Triangulenium Dye. *ACS Omega* **2019**, *4*, 5, 8381-8389, https://doi.org/10.1021/acsomega.9b00795

12. Clarke, J. S.; Achterberg, E. P.; Rerolle, V. M.; Abi Kaed Bey, S.; Floquet, C. F.; Mowlem, M. C. Characterisation and deployment of an immobilised pH sensor spot towards surface ocean pH measurements. *Anal Chim Acta* **2015**, *897*, 69-80, https://doi.org/10.1016/j.aca.2015.09.026

13. Larsen, M.; Borisov, S.M.; Grunwald, B.; Klimant, I.; Glud, R.N. A simple and inexpensive high resolution color ratiometric planar optode imaging approach: Application to oxygen and pH sensing. *Limnol. Oceanogr. Methods* **2011**, *9*, 348–360. https://doi.org/10.4319/lom.2011.9.348.

14. Schroder, C. R.; Weidgans, B. M.; Klimant, I. pH fluorosensors for use in marine systems. *Analyst* **2005**, *130*, 6, 907-16, https://doi.org/10.1039/b501306b
